# A Systematic Genetic Screen to Dissect the MicroRNA Pathway in *Drosophila*

**DOI:** 10.1534/g3.112.002030

**Published:** 2012-04-01

**Authors:** Sigal Pressman, Catherine A. Reinke, Xiaohong Wang, Richard W. Carthew

**Affiliations:** Department of Molecular Biosciences, Northwestern University, Evanston, Illinois 60208

**Keywords:** microRNA, miRNA, *Drosophila*, Dicer, Ago

## Abstract

A central goal of microRNA biology is to elucidate the genetic program of miRNA function and regulation. However, relatively few of the effectors that execute miRNA repression have been identified. Because such genes may function in many developmental processes, mutations in them are expected to be pleiotropic and thus are discarded in most standard genetic screens. Here, we describe a systematic screen designed to identify all *Drosophila* genes in ∼40% of the genome that function in the miRNA pathway. To identify potentially pleiotropic genes, the screen analyzed clones of homozygous mutant cells in heterozygous animals. We identified 45 mutations representing 24 genes, and we molecularly characterized 9 genes. These include 4 previously known genes that encode core components of the miRNA pathway, including *Drosha*, *Pasha*, *Dicer-1*, and *Ago1*. The rest are new genes that function through chromatin remodeling, signaling, and mRNA decapping. The results suggest genetic screens that use clonal analysis can elucidate the miRNA program and that ∼100 genes are required to execute the miRNA program.

MicroRNAs (miRNA) are recognized to be a fundamental class of regulatory molecules operating in plants and animals. A central goal of miRNA biology is to elucidate the genetic program of miRNA function and regulation. Many genes important for miRNA biogenesis and action have been isolated by candidate gene approaches, from biochemical inferences, and by genetic screens in cells and model organisms (Carthew and Sontheimer 2009; Czech and Hannon 2011). These approaches have been very successful in discovering key molecules in the miRNA pathway, but they have been less successful in identifying factors downstream of the pathway and regulators of the pathway. We reasoned that downstream factors and regulators of the miRNA pathway would function in multiple processes affecting gene expression and that mutations in these genes would be pleiotropic and thus discarded from most screens. We therefore designed a systematic mutagenesis screen for genes required for miRNA-mediated silencing that included clonal analysis of gene function. The results of the screen, representing 40% of the *Drosophila* genome, are described here.

When a miRNA is transcribed from the genome, the resulting primary or pri-miRNA transcript extends both 5′ and 3′ from the miRNA sequence, and two sequential processing steps trim the transcript into the mature miRNA (Bartel 2009). Processing depends on the miRNA sequence folding into a stem-loop structure. The first processing step, which occurs in the nucleus, excises the stem-loop from the remainder of the transcript to create a pre-miRNA product. For most pri-miRNAs, the RNase III enzyme Drosha carries out this cleavage reaction (Lee *et al.* 2003). Drosha requires a protein cofactor for efficient and precise processing (Gregory *et al.* 2004). In *Drosophila*, the cofactor Pasha contains two dsRBD domains and stably associates with Drosha to form the Microprocessor complex (Denli *et al.* 2004). However, this is not the only way to produce pre-miRNAs in animals. An alternative pathway uses splicing of transcripts to liberate introns that precisely mimic the structural features of pre-miRNAs. These mirtrons then enter the miRNA processing pathway without the aid of the Microprocessor (Martin *et al.* 2009; Ruby *et al.* 2007). The second processing step excises the terminal loop from the pre-miRNA stem to create a mature miRNA duplex of approximately 22 basepairs in length. The canonical Dicer RNase III carries out this cleavage reaction in the cytoplasm (Kim 2005). It is aided by the associated activity of a dsRBD-domain protein, Loquacious in *Drosophila* (Forstemann *et al.* 2005; Saito *et al.* 2005).

The mature miRNA duplex is a short-lived entity; it is rapidly unwound when it associates with an Argonaute (Ago) protein (Czech and Hannon 2011). Unwinding is accompanied by differential strand retention; one strand is retained by the Ago protein while the other strand is lost. Although either strand can become stably associated with Ago proteins, the more commonly associated strand is called the miRNA strand, and the other strand is called the miRNA^*^ strand (Kim 2005). The Ago-miRNA (miRISC) complex is also associated with the protein GW182 (Liu *et al.* 2005a; Meister *et al.* 2005; Till *et al.* 2007). In *Drosophila*, GW182 associates with the miRNA effector protein Ago1, and functional analysis indicates that GW182 is both necessary and sufficient for miRNA-bound Ago1 to silence gene expression (Behm-Ansmant *et al.* 2006; Eulalio *et al.* 2008).

With few exceptions, miRNA-binding sites in RNA transcripts lie in the 3′ UTR and are usually present in multiple copies (Bartel 2009). Most miRNAs bind with mismatches and bulges, although a key feature of recognition involves Watson-Crick base pairing of miRNA nucleotides 2–8, representing the seed region. The mechanisms by which miRISC represses gene expression are complex and involve both protein translation and mRNA stability [reviewed in Carthew and Sontheimer (2009), Filipowicz *et al.* (2008), and Huntzinger and Izaurralde (2011)]. Some studies have found evidence for miRISC-repressed translational initiation whereas other studies have found evidence for repression of post-initiation processes. Currently, there are three competing models for how miRISC represses initiation. One model proposes that miRISC competes with eIF4E for binding to the mRNA 5′ cap structure and thereby blocks 40S subunit recruitment. A second model has proposed that miRISC stimulates deadenylation of the mRNA tail. In this model, translation is repressed because the cap and tail of the deadenylated mRNA are unable to circularize. A third model has proposed that miRISC blocks association of the 60S subunit with the 40S pre-initiation complex.

Transcript stability can also be affected by miRISC (Carthew and Sontheimer 2009; Filipowicz *et al.* 2008; Huntzinger and Izaurralde 2011). For many miRNA-target interactions, there is a significant reduction in mRNA abundance due to an increase in mRNA degradation. This increased degradation is the result of deadenylation, decapping, and exonucleolytic digestion of the mRNA. A critical question is whether degradation is a consequence of a primary effect on translation. Evidence suggests that degradation can be uncoupled from translation (Huntzinger and Izaurralde 2011). At present it is unclear why some targets are degraded and others are not. It has been suggested that the number, type, and position of mismatches in the miRNA/mRNA duplex play an important role in triggering degradation or translation arrest (Aleman *et al.* 2007).

A comprehensive screen for components of the miRNA pathway in *C. elegans* has been performed (Parry *et al.* 2007). In *Drosophila*, however, systematic mutagenesis screens identifying novel genes in the miRNA pathway have not yet been reported. One screen for components of the miRNA pathway identified alleles of *Dicer-1*, *Pasha*, and *Drosha* (Smibert *et al.* 2011), and mutations in *Dicer-1*, *Loquacious*, *Ago1*, and *Pasha* have been isolated as a result of targeted mutagenesis (Forstemann *et al.* 2005; Kataoka *et al.* 2001; Lee *et al.* 2004; Martin *et al.* 2009). A comprehensive RNAi screen of the miRNA pathway was performed in S2 cell culture, and positive hits were enriched for genes acting in chromatin remodeling, RNA metabolism, and ubiquitin-proteasome processes (Zhou *et al.* 2008). Together these approaches have implicated ∼100 genes in miRNA function. However, the absence of several known genes from the screens suggest that particular screen methodologies can bias the identification of genes in unpredictable ways and that there are more unidentified genes that act in the *Drosophila* miRNA pathway.

We conducted a large-scale screen of chemical- and transposon-induced mutations to assess the function of 40% of the *Drosophila* genome, including early essential genes and genes with pleiotropic phenotypes. We sought to create allelic series in known genes and to identify new genes associated with miRNA function. We anticipated that this approach would provide an estimate of the total number of genes required for miRNA silencing. Our screen assayed clones of homozygous mutant cells in the compound eye of otherwise heterozygous individuals. We isolated mutations representing 24 genes, 9 of which we identified molecularly, implicating previously identified genes and revealing new factors in silencing regulation.

## MATERIALS AND METHODS

### Transgenes

The 3′UTR of the *Bearded* (*Brd*) gene plus an additional 134 bp on its 3′ flank was inserted downstream of *eGFP* coding sequence. This was then inserted into the pGMR plasmid to generate *GMR > eGFP*::*Brd*. We deleted the vector’s mini-*white* transformation marker gene so that the transgenic animals would have nonpigmented eyes. This allowed us to detect GFP fluorescence in eyes of transformed animals. A *GMR > eGFP*::*Brd* fragment, which had its *miR-4* binding site seeds changed from AGCTTTA to ATAGGGA, was cloned into a pPelican vector with its mini-*white* marker gene deleted. This generated *GMR > eGFP*::*Brdmut*. Transformant lines were generated by standard means and were identified by GFP fluorescence in eyes. The *tub > eGFP*::*Hid*, *arm > LacZ*::*E(spl)m8*, and *tub > eGFP*::*2x(miR-7)* transgenes were described previously (Brennecke *et al.* 2003; Lai and Posakony 1997; Li and Carthew 2005).

### *Drosophila* stocks

#### For mutagenesis:

*yellow (y) white (w)*, *eyFLP* ; *FRT42D* (isogenized second chromosome)
*y w*, *eyFLP* ; *FRT82B* (isogenized third chromosome)

#### For screening:

*y w*, *eyFLP* ; *FRT42D*, *P{y+}* ; *GMR > GFP*::*Brd*
*y w*, *eyFLP* ; *GMR > eGFP*::*Brd*, *FRT82B ebony (e) / TM3*, *e*

#### For testcrosses:

*y w*, *eyFLP* ; *FRT42D* ; *arm > LacZ*::*E(spl)m8*
*y w*, *eyFLP* ; *FRT42D*, *arm > LacZ* ; *tub > eGFP*::*Hid*
*y w*, *eyFLP* ; *FRT42D*, *GMR > myr-RFP* ; *GMR > GFP*::*Brd*
*y w*, *eyFLP*; *FRT42D*, *arm > LacZ*; *GMR > GFP > GFP*::*Brd*
*y w*, *eyFLP* ; *GMR > GFP*::*Brdmut*, *FRT42D*, *GMR > myr-RFP*
*y w*, *eyFLP* ; *GMR > eGFP*::*Brd*, *FRT82B*, *GMR > myr-RFP / TM6B*
*y w*, *eyFLP* ; *GMR > eGFP*::*Brdmut* ; *FRT82B*, *GMR > myr-RFP / TM6B*
*y w*, *eyFLP* ; *arm > LacZ*::*Brd* ; *FRT82B / TM6B*
*y w*, *eyFLP* ; *arm > LacZ*::*E(spl)m8*, *FRT82B / TM6B*

#### For eye phenotype analysis:

*y w*, *eyFLP* ; *FRT42D*, *GMR > Hid / CyO*
*y w*, *eyFLP* ; *FRT82B*, *GMR > Hid / TM6B*

#### For lethal phase analysis:

*y w*, *eyFLP*; *FRT82B / TM6B*, *dfd-YFP w+*

### Mutagenesis

Three-day-old *y w eyFLP* ; *FRT* males were starved for two hours before feeding overnight on ethyl methanesulfonate (EMS) in 1% sucrose, as described (Lee *et al.* 2004; Lewis and Bacher 1968). Males were allowed to recover for a few hours, and were then crossed *en masse* to *y w eyFLP* ; *FRT* females carrying the *GMR > eGFP*::*Brd* transgene. The female’s *FRT* chromosome contained either a dominant (*P{y+}*) or recessive (*e*) marker that enabled us to select in subsequent generations for animals who did not carry the marker, and hence, carried a mutagenized *FRT* chromosome. F1 males were examined under a dissecting fluorescence microscope for variegated GFP fluorescence in their compound eyes. Each positive male was backcrossed to *y w eyFLP* ; *FRT* females carrying the *GMR > eGFP*::*Brd* transgene. F2 males bearing the genotype *y w*, *eyFLP* ; *FRT*, *mutation* (^*^) / *FRT* were also examined under the microscope for variegated GFP fluorescence. Those males with variegation were crossed to *y w*, *eyFLP* ; *FRT / Balancer* females. F3 animals bearing the genotype *y w*, *eyFLP* ; *FRT*, ^*^ / *FRT* were assayed for variegated fluorescence, and their *y w*, *eyFLP* ; *FRT*, ^*^ / *Balancer* sibs were self-crossed to establish balanced stocks. An example of the screen done on 2R is shown in supporting information, Figure S1.

To screen lethal piggyBac and P insertional mutations, we utilized two collections from the Drosophila Genetic Resource Center (DGRC, Kyoto, Japan), where insertion mutations had been recombined on *FRT* chromosomes (Chen *et al.* 2005; Schuldiner *et al.* 2008). Each line was crossed to *y w*, *eyFLP* ; *FRT* females containing *GMR > GFP*::*Brd*. F1 animals were assayed for variegated eye disc fluorescence, and any positive hits were re-assayed.

### Mapping

We assumed that the mutation causing variegated GFP fluorescence was functionally linked to organismal lethality. Therefore, we mapped the positions of lethal mutations on the mutagenized chromosomes by calculating the frequency of recombination between the lethal mutation and a series of *mini w+*-marked *P*-element insertions located at precise intervals along the chromosome as previously described (Zhai *et al.* 2003). To test our assumption regarding linkage between lethality and GFP expression, viable recombinant progeny from each mapping cross were examined for the GFP phenotype. A strict correlation between viability and lack of a GFP phenotype confirmed this assumption. Once loci had been mapped to a chromosomal interval, we performed fine-scale mapping by crossing mutant lines to flies carrying annotated deficiencies within the chromosomal interval. Complementation/noncomplementation of mutant lethality was used to narrow the loci to as fine a scale as possible. Complementation/noncomplementation of mutant lethality by alleles of known miRNA pathway components was also used to identify new alleles of miRNA pathway component genes isolated during this screen.

### Molecular mapping

*y w*, *eyFLP* ; *FRT*, ^*^ / Balancer flies were crossed to the *y w*, *eyFLP* ; *FRT* isogenized parental strain to generate heterozygous flies. Genomic DNA was prepared from these flies for the amplification and sequencing of individual genes. Genomic DNA was prepared by homogenizing adult flies in 50 µl 10 mM Tris-Cl pH 8.2, 1 mM EDTA, 25 mM NaCl, 200 µg/ml proteinase K, and incubating at 37° for 30 min followed by incubation at 95° for 2 min to heat inactivate proteinase K (Gloor *et al.* 1993). For sequencing, both strands of candidate transcription units were sequenced to detect differences between parental and mutagenized DNA sequence.

### Complementation analysis

Mutant autosomes were placed into a background lacking *eyFLP*, and pairwise crosses were made between these mutant lines. F1 heterozygotes were examined for either lethality or changes in GFP fluorescence. Lethality or changes in GFP fluorescence indicated noncomplementing mutants, and they were classified as alleles. When available, we also tested allelic groups for complementation with known alleles of miRNA pathway genes.

### Analysis of eye development

*y w*, *ey*FLP ; *FRT*, *GMR > Hid / Balancer* animals were crossed to *y w*, *eyFLP* ; *FRT*, ^*^ / Balancer animals to generate *y w*, *ey*FLP ; *FRT*, ^*^ / FRT, *GMR > Hid* F1 adults. The *GMR > Hid* transgene drives apoptosis in all eye cells that do not recombine to become homozygous mutant. Remaining mutant eye cells developed into adult eyes that were examined using a Hitachi S-3400N-II scanning electron microscope (SEM). SEM specimens were prepared by freezing adults at −80° for 10 min and mounting them directly onto an aluminum specimen mount with Electrodag 502 conducting graphite paint (Ted Pella Inc.). Images were captured with PC-SEM software and transferred to Quartz PCI software.

### Determination of lethal phase

*w* ; *FRT*, ^*^ / Balancer males were mated to either *w* ; *Df / Balancer* or *w* ; *FRT*, ^*^(null) / Balancer females at 25°. Balancers carried *dfd > YFP* (Le *et al.* 2006) or *twi > GFP* to enable detection in embryos and larvae. Embryos were collected over 2 hr intervals on egg-laying plates, and those lacking *dfd > YFP* or *twi > GFP* fluorescence were selected and transferred to fresh plates. These were incubated at 25°, and the number of living animals was counted daily as well as the stage of development.

### *In situ* activity, immunohistochemistry, and Western analysis

Homozygous mutant clones were generated using the *FLP-FRT* technique (Xu and Rubin 1993). Wildtype *FRT* chromosomes carried either myristoylated RFP (*myr-RFP*) driven by the *GMR* promoter or *LacZ* driven by the ubiquitous *armadillo* (*arm*) promoter. These allowed us to mark the mutant cells by virtue of the absence of the marker. Eye discs were fixed in 4% paraformaldehyde on ice. After washing in PBT (PBS + 0.2% Triton X-100), immunostaining was performed (Li and Carthew 2005). We used anti-Ago1 (1:200 a gift from M. Siomi, Keio University, Tokyo) and anti-β-Galactosidase (1:750 a gift from G. Beitel, Northwestern University, IL), followed by Alexa 488- or 594-conjugated goat antibodies (1:200; Molecular Probes). Discs were mounted in Vectashield or 90% glycerol and visualized on a Zeiss LSM 510 laser scanning confocal microscope. To detect expression from the *arm > LacZ*::*E(spl)m8* transgene, we used X-gal activity staining of eye discs as described (Xu *et al.* 2000). Although this method did not allow us to mark the mutant clones, it was the only way to detect β-Galactosidase from the reporter as immunostaining was not sensitive enough (data not shown). Nevertheless, the strict correlation between mosaic mutant genotype and variegated X-gal staining supports the clonal nature of the staining pattern (N > 35 discs).

For Western blots, embryos were collected 16 hr after egg laying and were lysed in extraction buffer. After clarification, protein was loaded on an SDS-PAGE gel and blotted to nitrocellulose. Mouse anti-Ago1 (1:2000) and anti-α-tubulin (1:1000) were used to visualize proteins by ECL.

## RESULTS

### Screen design

**Figure 1:**
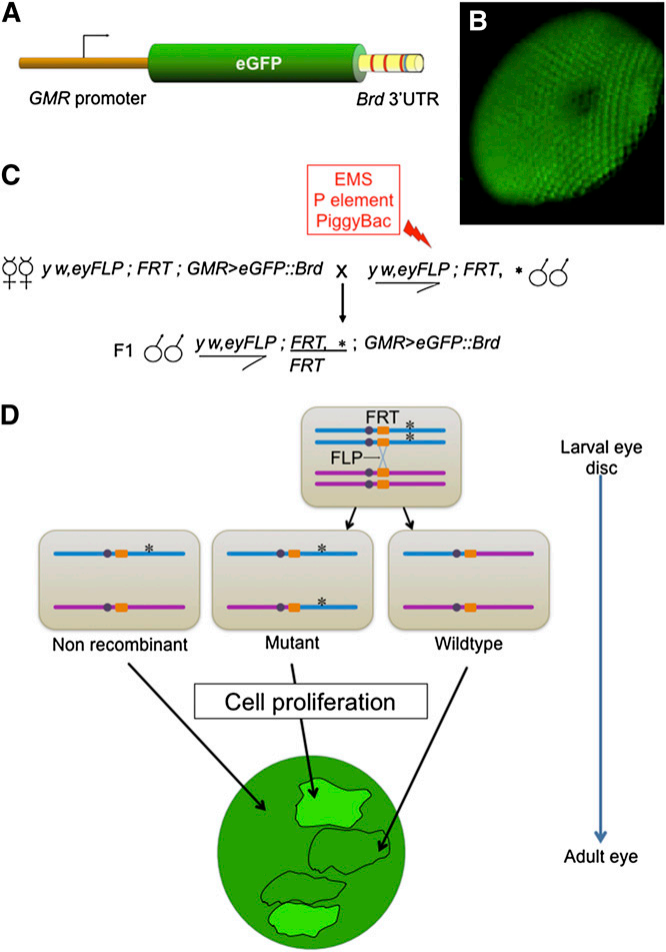
Design of the genetic screen. (A) Diagram of the *GMR > eGFP*::*Brd* transgene reporter. Binding sites for miR-4/-79 (red) and miR-7 (blue) are indicated in the *Brd* 3′UTR. (B) Fluorescence micrograph of the compound eye of an animal expressing one copy of the *GMR > eGFP*::*Brd* transgene. (C) Genetic scheme of the F1 mosaic screen. *, mutagenized chromosome; EMS, ethyl methanesulfonate; *eyFLP*, FLP recombinase transgene under *eyeless* promoter control (eye-specific); *FRT*, a FLP recombinase target site transgene (either FRT42D on 2R or FRT82B on 3R) located adjacent to the second and third chromosome centromeres; *w*, *white*; *y*, *yellow*. (D) Scheme of the assay in F1 animals. During the larval phase, FLP catalyzes chromosome crossover during mitosis of eye cells. Homozygous wildtype and mutant daughter cells are generated. Cells in which mitotic crossover has not occurred are heterozygous nonrecombinants. Further cell proliferation results in clones of mutant cells. Fluorescence from the reporter transgene is assayed in adult eyes, and mutant clones with impaired miRNA silencing display higher fluorescence than nonmutant neighboring regions.

Mutants were screened for defects in miRNA silencing by assaying a transgene reporter (Figure 1A). The reporter contains the eGFP coding sequence regulated by two distinct elements. The *GMR* transcription promoter limits eGFP expression to the compound eye of *Drosophila*. The 3′UTR from the *Bearded* (*Brd*) gene renders eGFP expression sensitive to miRNA repression. This UTR has been experimentally demonstrated to contain three binding sites for miR-4/miR-79 and one binding site for miR-7 (Lai *et al.* 2005). Mutation of the binding sites has been shown to derepress gene expression *in vivo* (Lai and Posakony 1997). Transgenic animals containing the *GMR > eGFP*::*Brd* transgene uniformly exhibited low but detectable green fluorescence in their compound eyes (Figure 1B).

To discover new genes required for miRNA silencing, we used a genetic mosaic strategy in which we examined clones of homozygous mutant eye cells in otherwise heterozygous animals (Figure 1, C and D). This approach enabled us to recover mutations that otherwise were homozygous lethal to the organism. FLP recombinase was expressed in larval eye cells and catalyzed DNA recombination between FRT sites located on two sister chromosomes (Newsome *et al.* 2000; Stowers and Schwarz 1999). The resulting recombined chromosomes segregated during mitosis such that one daughter cell inherited both copies of one sister chromosome, and the other daughter cell inherited copies of the other sister chromosome. Subsequent cell proliferation produced clones of eye cells with the same inherited chromosomes. This approach allowed us to assay mutations that are organismal-lethal but not mutations that are cell-lethal as no clones would be produced from cell-lethal mutants. To identify mutants affecting miRNA silencing, we screened mutant eyes for variegated GFP fluorescence, which was caused by altered *GMR > eGFP*::*Brd* expression in homozygous mutant clones.

Because recombination is limited to a single arm of each chromosome, we performed the screen independently on the right arms of chromosomes 2 and 3. These represent 40% of the genome. Our rationale for choosing these arms was that they contained *Ago1*, *Drosha*, *Pasha*, and *Dicer-1* genes, and thus, we expected to isolate mutations in these genes if the screen was successful. Mutagenesis was performed in two different ways. We screened a collection of lethal *P*-element and PiggyBac-element insertional mutations that have been mapped to the genome (Schuldiner *et al.* 2008). We also induced mutations by EMS, as the efficiency of mutagenesis enables screening to apparent saturation. The EMS mutagenesis procedure induces an average of 0.65 lethal mutations per chromosome arm (Nüsslein-Volhard *et al.* 1984), and given that we screened a total of 56,000 mutagenized chromosome arms, 29,900 lethal mutations were screened for phenotypes. There are ∼3600 essential *Drosophila* genes (Spradling *et al.* 1999), with roughly 40% (∼1440) on the two chromosome arms that we screened. We expected to observe an average of 21 (29,900/1440) mutations per gene, with at least one mutation in more than 99.9% of all genes.

### Overview of screen results

**Figure 2:**
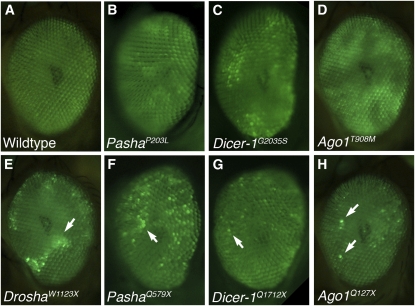
Mutant phenotypes. Fluorescence from *GMR > eGFP*::*Brd* in eyes that are completely wildtype (A), or that contain clones that are homozygous mutant for missense alleles in *Pasha* (B), *Dicer-1* (C), and *Ago1* (D), or that are homozygous mutant for nonsense alleles in *Drosha* (E), *Pasha* (F), *Dicer-1* (G), and *Ago1* (H). Alleles are indicated. Note the large patches of brighter fluorescence in missense mutant eyes. The bright patches in nonsense mutant eyes (E–H) are much smaller, and several are highlighted by arrows. The patches in *Dicer-1* and *Ago1* mutants are very small, encompassing one or two ommatidia.

We expected that mutations in genes that promoted miRNA-mediated silencing of the eGFP reporter would cause enhanced fluorescence within mitotic eye clones. Over 950 F1 mutants with variegated eye fluorescence were identified from the screen. Of those, only 73 mutants were recovered in the F3 generation as balanced stocks. All of the mutations were homozygous lethal. A number of the mutations that were recovered in the F3 generation had weakly penetrant and expressive phenotypes, and thus we did not consider them for further analysis. The remaining 45 highly penetrant and expressive mutations exhibited robust variegation of the eGFP reporter transgene in mosaic eyes (Figure 2).

Complementation tests allowed assignment of the 45 mutations to 24 loci. The mean number of alleles per locus was 1.9, a number far lower than the expected average frequency of 21 predicted by a Poisson distribution. Several factors may have resulted in this discrepancy. The observed 8% frequency of mutant recovery from our F1 mosaic screen is typical when phenotypes are assayed in the adult eye (Lee *et al.* 2004). The reason for the low recovery has been speculated to be due to mosaicism of mutagenized cells within F1 animals, making recovery of the mutation in subsequent generations difficult (Jenkins 1967; Karim *et al.* 1996). Nevertheless, based on the observed allele frequency, we estimate that 85% of loci involved in miRNA silencing were identified in the screen.

### Gene identification reveals new pathways

To begin to define the molecular functions of genes identified in the screen, we mapped representative mutations and molecularly identified nine of the genes (Table 1). Four of the identified genes *(Ago1*, *Dicer-1*, *Drosha*, and *Pasha*) were previously implicated in miRNA silencing in *Drosophila*. New mutations in these genes were identified by the following critera: (i) they failed to complement various deficiencies or other alleles that uncovered each of these genes, or (ii) a single residue in gene coding sequence was changed in each mutant that either created a nonsense codon or altered an invariant amino acid. New genes were also revealed by our analysis. One gene (*Dcp1*) encodes a subunit of the mRNA decapping enzyme, which has been hypothesized as an effector of miRNA silencing (Behm-Ansmant *et al.* 2006). Three genes (*grappa*, *Bap55*, and *domino*) encode chromatin-remodeling factors. One gene (*Syndecan*) encodes a transmembrane coreceptor present on all adherent cells that transduces signals from the extracellular matrix (ECM) to cytoplasm, regulating diverse cell activities (Spring *et al.* 1994). The extracellular domain contains attachment sites for heparan sulfate polysaccharide chains that mediate interactions with ECM components, heparin-sulfate growth factors, cell adhesion molecules, lipases, chemokines, cytokines, and their receptors (Couchman 2003). Thus, a diversity of factors were identified from the genetic screen.

**Table 1:** Genes and molecular aberrations identified from the screen

Gene	Allele	Molecular Mutation	Pass Secondary*^a^*
Drosha	Q884X	Gln884→Stop ; CAA→TAA	Yes
	Q938X	Gln938→Stop ; CAG→TAG	Yes
	W1123X	Trp1123→Stop ; TGG→TGA	Yes
Pasha	R59X	Arg59→Stop ; CGA→TGA	Yes
	Q83X	Gln83→Stop ; CAG→TAG	Yes
	P203L	Pro203→Leu ; CCC→CTC	Yes
	Q394X	Gln394→Stop ; CAA→TAA	Yes
	Q579X	Gln579→Stop ; CAG→TAG	Yes
	A13	Unknown	Yes
Dicer-1	K43X	Lys43→Stop ; AAG→TAG	Yes
	W94X	Trp94→Stop ; TGG→TGA	Yes
	Q396X	Gln396→Stop ; CAG→TAG	Yes
	Q770X	Gln770→Stop ; CAA→TAA	Yes
	Q991X	Gln991→Stop ; CAA→TAA	Yes
	Q1233X	Gln1233→Stop ; CAG→TAG	Yes
	Q1712X	Gln1712→Stop ; CAA→TAA	Yes
	G2035S	Gly2035→Ser ; GGC→AGC	Yes
Ago1	Q127X	Gln127→Stop ; CAG→TAG	Yes
	D743N	Asp743→Asn ; GAT→AAT	Yes
	E808K	Glu808→Lys ; GAA→AAA	Yes
	R839X	Arg839→Stop ; CGA→TGA	Yes
	W894X	Trp894→Stop ; TGG→TGA	Yes
	T908M	Thr908→Met ; ACG→ATG	Yes
	R937C	Arg937→Cys ; CGT→TGT	Yes
	J04	Unknown	Yes
Bap55	LL05955	2R:13317566(−) ; CDS − V_234_ ▽ K_235_*^b^*	Yes
Dcp-1	EY16846	2R:19777750(−) ; 5′UTR − A_34_ ▽ A_35_	Yes
Domino	LL05537	2R:17211471(+) ; intron1 − T_343_ ▽ T_344_	Yes
Grappa	Q210X	Gln210→Stop ; CAG→TAG	Yes
Syndecan	LL00212	2R:17367838(−) ; 5′UTR − A_149_ ▽ C_150_	Yes

a Mutations that were tested to affect other reporters as a secondary test for miRNA specificity are indicated.

b Insertions of piggyBac or P elements are designated by a ▽ symbol. Insertion point is indicated by the genome sequence position (version FB2011_08, released Sept 2, 2011), the region of the gene, and the base or amino acid sequence position within that gene region.

The other 15 complementation groups that we isolated but did not identify were all generated by EMS mutagenesis. Five of them were tested for their effects on other miRNA sensors, and 4 of them also affect other sensors, suggesting that they are more generally involved in miRNA regulation.

### Molecular characterization of mutations in core miRNA pathway genes

We sequenced the mutant protein-coding sequence for each allele of *Ago1*, *Dicer-1*, *Drosha*, and *Pasha*, and except for one *Ago1* and *Pasha* allele, each mutant contained a single change in the coding sequence that altered the polypeptide product (Table 1 and Figure S2). The transcription units of the *Ago1^J04^* and *Pasha^A13^* alleles were completely sequenced, but no mutations were identified. We presume that the responsible mutations might lie in regulatory sequences outside of the units. All genes had at least one mutant allele that contained a nonsense codon, and these alleles we consider presumptive null. In addition, missense mutants were isolated for *Dicer-1*, *Pasha*, and *Ago1*. The *Pasha* mutation changes an invariant proline in the WW domain, which is conserved among all known Pasha orthologs. The precise function of the Pasha WW domain remains unknown. The *Dicer-1* mutation changes an invariant glycine that is part of the signature motif of RNase III domains, and it is immediately adjacent to a catalytic carboxylate in the second RNase III domain (Macrae *et al.* 2006). It is likely that the change would severely impair catalytic activity of the enzyme. The four *Ago1* mutations altered conserved amino acids within the protein’s Piwi domain (Figure S3). The Piwi domain of *Drosophila* Ago1 is a RNase H–like endonuclease that cleaves a single phosphodiester bond in the target RNA backbone if it is perfectly complementary to an associated miRNA (Miyoshi *et al.* 2005). There are two sequence motifs, a GxDV and an RDG motif, within the Piwi domain that are highly conserved in eukaryotic Ago proteins. The two aspartate residues of these motifs are structurally equivalent to two aspartate residues that coordinate a metal ion at the catalytic core of RNase H (Parker *et al.* 2004; Song *et al.* 2004; Yang and Steitz 1995). A third coordinating carboxylate varies in its position within the active site of RNase H. Studies have determined a histidine residue to be the predominant third residue of Ago necessary for catalysis (Rivas *et al.* 2005). One of the mutations identified in our screen, *Ago1^E808K^*, changes an invariant glutamate residue that is conserved in all sequenced eubacterial and eukaryotic Piwi domains. Importantly, this residue is located 13 residues from the RDG motif, and the homologous residue in crystallized eubacterial Ago proteins is proximate to the active site. Another mutation identified in our screen, *Ago1^R937C^*, changes an arginine residue located only five residues from the coordinating histidine in Ago1, and the homologous residue in a crystallized eukaryotic Ago protein is critical for the 5′ end of guide RNA to bind to the protein (Boland *et al.* 2011). Mutation of this residue abolishes guide RNA binding, and in *Drosophila* Ago1, abolishes miRNA binding.

In the case of *Ago1*, we observed complex complementation between an existing mutation, *l(2)k08121*, and all of our mapped EMS alleles of *Ago1*. *l(2)k08121* partially or fully complemented the lethality associated with all of our alleles. Moreover, *l(2)k08121* partially complemented the lethality associated with the deficiency *Df(2R)CX1* that completely uncovers the *Ago1* locus. In contrast, all our new *Ago1* alleles completely failed to complement the deficiency. We ascribe these differences to the hypomorphic nature of *l(2)k08121*. There are four *Ago1* transcripts, and the *l(2)k08121* mutation is caused by a *P*-element insertion in the second intron of the C and D transcripts (Grimaud *et al.* 2006; Kataoka *et al.* 2001; Roch *et al.* 1998). We interpret this to mean that the P allele disrupts some but not all *Ago1* mRNA isoforms, and thus the P alleles can complement alleles that affect all isoforms (the deficiency and all of our point mutations).

**Figure 3:**
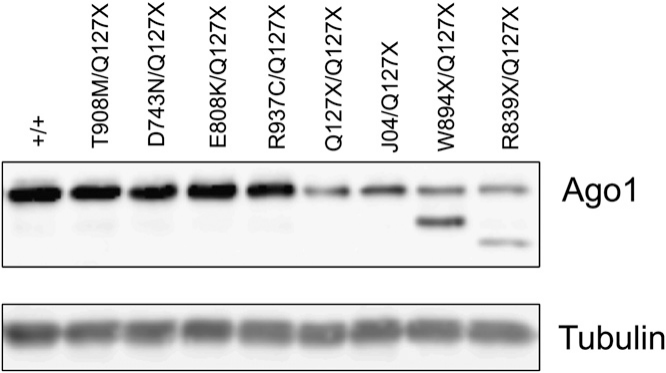
Ago1 protein produced by *Ago1* alleles. Western blots of Ago1 protein from 16-hour zygotic mutant embryos. Wildtype protein was derived from the parental fly line used for mutagenesis. Note the reduced abundance of full-length protein in nonsense mutants. α-tubulin was used as a loading/blotting control.

We used a high-affinity antibody against Ago1 to determine whether the *Ago1* mutants produced protein. We performed Western blots on proteins from 16-hr-old zygotic mutant embryos. Nonsense mutants showed a strong reduction in the level of full-length protein (Figure 3). We surmise that the remaining full-length protein was maternally loaded from the heterozygous mothers for the following reasons. These mutants showed greater abundance of full-length Ago1 protein if they were harvested at earlier stages of embryonic development, consistent with a gradual decay in protein coming from the eggs (data not shown). Moreover, immunohistochemistry of mosaic eye discs confirmed that the nonsense alleles were protein-null because no fluorescence was detected within mutant cells (Figure S4). For two nonsense mutants, smaller Ago1 protein products were detected in the Western blot that were consistent in size with being generated by truncated protein synthesis (Figure 3). For the four missense mutants, Ago1 abundance and size were normal. These results suggest that the missense mutants make full-length Ago1 protein, but the protein is defective for activity.

### Core pathway gene function

**Figure 4:**
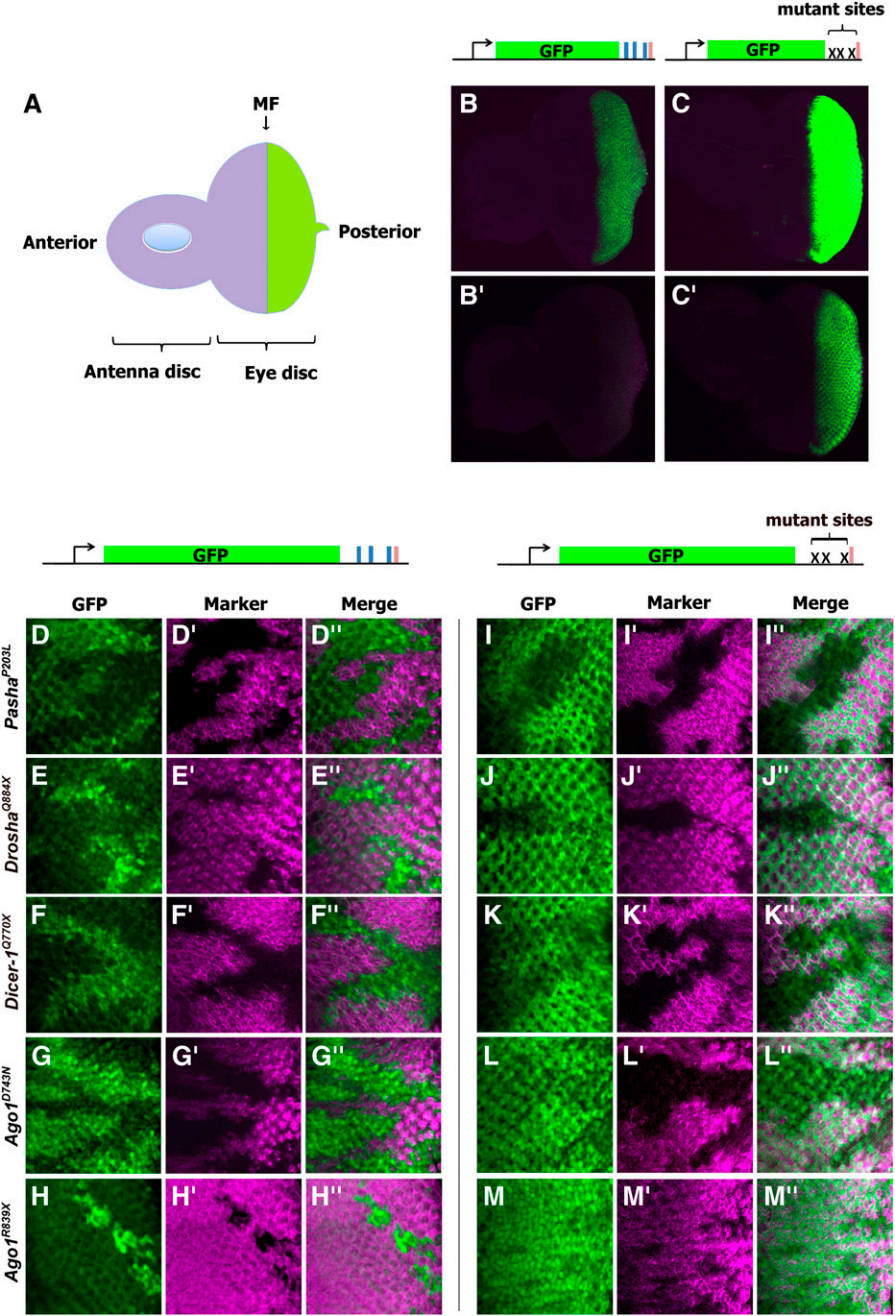
Mutant cells derepress the *GMR > eGFP*::*Brd* reporter in the larval eye. (A) Scheme of third instar larval eye-antennal discs. Posterior to the morphogenetic furrow (MF), cells initiate expression of the reporter gene. (B) A disc from a *GMR > eGFP*::*Brd* animal. (C) A disc from a *GMR > eGFP*::*Brdmut* animal. The exposure settings of discs in (B) and (C) are equivalent. (B′, C′) The same discs are shown where the exposure settings are adjusted lower such that the fluorescence from *GMR > eGFP*::*Brdmut* is not saturating. (D–H) Magnified regions of posterior eye discs from *GMR > eGFP*::*Brd* animals that contain clones of mutant *Pasha* (D), *Drosha* (E), *Dicer-1* (F), or *Ago1* (G, H) cells. Shown is eGFP fluorescence in green. (D′–H′) The marker expression (purple) is only detected in wildtype and nonrecombinant cells. Cells not expressing the marker are homozygous mutant. (D′′–H′′) The merged images for eGFP and the marker. (I–M) Magnified regions of posterior eye discs from *GMR > eGFP*::*Brdmut* animals that contain clones of mutant *Pasha* (I), *Drosha* (J), *Dicer-1* (K), or *Ago1* (L, M) cells. Shown is eGFP fluorescence in green. (I′–M′) The marker expression (purple) is only detected in wildtype and nonrecombinant cells. Cells without the marker are homozygous mutant. (I′′–M′′) The merged images for eGFP and marker. Alleles are as indicated.

We examined the effects of mutations on reporter expression in the developing larval eye by identifying the mutant cells using a genetic marker. The *GMR > eGFP*::*Brd* reporter was expressed in larval eye cells located posterior to the morphogenetic furrow, a moving wave of cellular differentiation (Figure 4A). We also examined expression of a different reporter transgene, *GMR > eGFP*::*Brdmut*, which has its miR-4/-79 binding sites mutated in the *Brd* 3′UTR. As expected, the level of eGFP expression in the larval eye was much greater from this mutant transgene when compared with *GMR > eGFP*::*Brd* (Figure 4, B and C).

We next generated mutant clones that were identified by the lack of expression of a marker transgene. Such marked clones mutant for *Pasha*, *Drosha*, *Dicer-1*, and *Ago1* (nonsense or missense) had increased levels of *GMR > eGFP*::*Brd* reporter expression (Figure 4, D–H, Figure S5, Figure S6, Figure S7, Figure S8, and Figure S9.). In contrast, mutant clones did not show increased levels of *GMR > eGFP*::*Brdmut* reporter expression (Figure 4, I–L), indicating that the repressive effects of these genes on the *GMR > eGFP*::*Brd* reporter are mediated through miRNA binding. Interestingly, the mutant clones showed weaker expression from *GMR > eGFP*::*Brdmut*, suggesting that the four genes activate the *GMR > eGFP*::*Brd* reporter in parallel to their direct repression of the reporter mediated by miRNA binding. This activation is likely indirect and mediated through miRNA regulation of transgene repressors.

**Figure 5:**
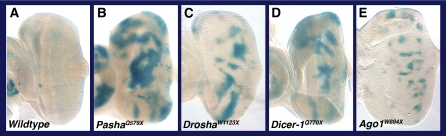
Mutant cells derepress the *LacZ*::*E(spl)m8* reporter transgene in the larval eye. (A) Wildtype, showing very low level of expression in the eye as seen after X-Gal staining (blue). Expression is strongly induced in eyes with mutant clones in *Pasha* (B), *Drosha* (C), *Dicer-1* (D), and *Ago1* (E). Specific alleles are indicated.

To determine whether the mutants affected repression of multiple miRNA targets, we assayed the expression of a LacZ reporter transgene that was under control of the *E(spl)m8* 3′UTR. This UTR contains two binding sites for miR-2, which exert strong repression in the developing larval eye (Lai and Posakony 1997). Mosaic clones mutant for *Ago1*, *Dicer-1*, *Drosha*, and *Pasha* showed significant reporter gene derepression when compared with the very weak expression in neighboring wildtype cells (Figure 5). Thus, the four genes, as expected, are required for repression of multiple targets by multiple miRNAs.

It has been shown that if target RNAs are perfectly complementary to a miRNA, then Ago proteins can slice (cleave) the target RNA providing they contain a fully functional Piwi domain (Miyoshi *et al.* 2005). We wondered whether the amino acid substitutions in the Ago1 Piwi domain that we isolated might impair its Slicer activity. Therefore, we expressed an eGFP reporter that contained two perfect binding sites for the miRNA miR-7 in its 3′UTR. When we made mutant clones of *Ago1* missense alleles, we found the reporter was derepressed to the same extent as *Ago1* null alleles (Figure S10). Thus, the Piwi mutations affect repression by perfect and imperfect miRNA interactions alike.

**Figure 6:**
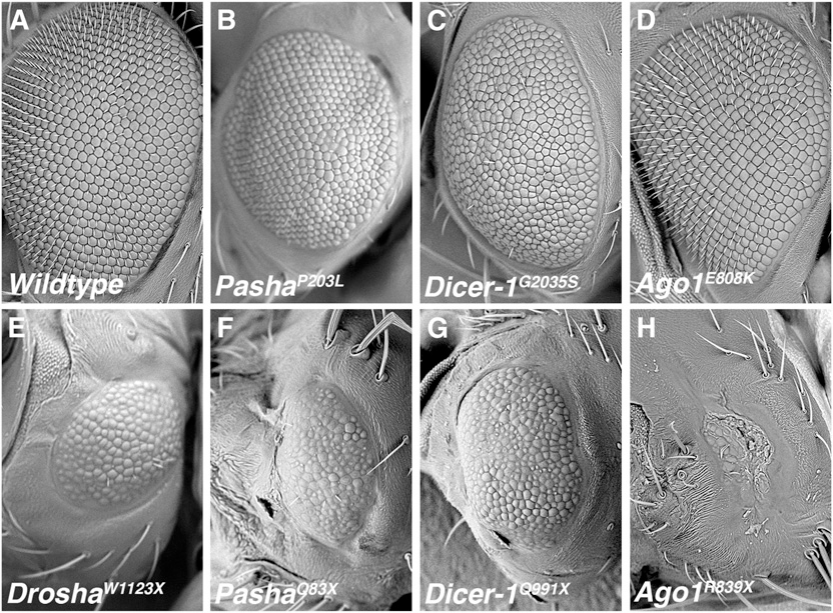
Formation of the compound eye requires miRNA pathway genes. Scanning electron micrographs of eyes in which the entire eye tissue is wildtype (A), or homozygous mutant for missense alleles in *Pasha* (B), *Dicer-1* (C), and *Ago1* (D), or homozygous mutant for nonsense alleles in *Drosha* (E), *Pasha* (F), *Dicer-1* (G), and *Ago1* (H). Specific alleles are indicated.

MicroRNAs are essential for organismal viability, affecting numerous developmental and physiological pathways. In *Drosophila*, miRNAs are required for germ cell development, cell survival, differentiation, and morphogenesis (Bushati and Cohen 2007). We sought to examine the roles of the four core pathway genes in eye development by generating eyes that were completely homozygous mutant. This was done by using a cell death gene to kill all wildtype and nonrecombinant cells in the developing eye (Stowers and Schwarz 1999). The adult compound eyes of such mutants were disrupted to varying degrees. Missense mutations in *Pasha* and *Dicer-1* resulted in mispatterned and smaller eyes (Figure 6, A–C). Three of the *Ago1* missense mutants exhibited very mild mispatterning (Figure 6D), and the fourth mutant, *Ago1^R937C^*, had a small necrotic eye (data not shown). We also examined various nonsense mutants for eye development phenotypes (Figure 6, E–H). *Drosha*, *Pasha*, and *Dicer-1* nonsense mutants had small mispatterned eyes, whereas for *Ago1* nonsense mutants, the size of the eyes was greatly reduced. In the case of the *Ago1^W894X^* mutant, there was virtually no eye to be found (data not shown).

**Figure 7:**
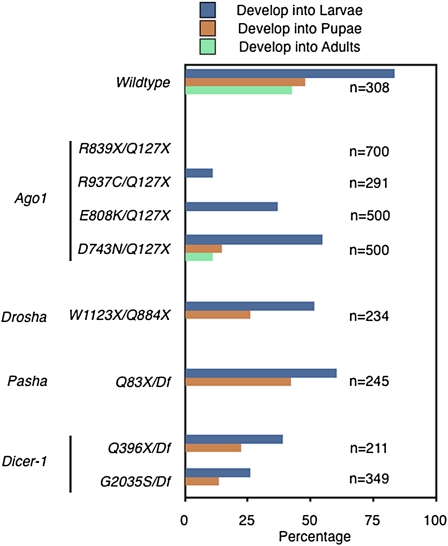
Lethal phase analysis of pathway mutants. Zygotic mutant embryos were collected and raised at 25°, monitoring the number of animals that survived into the different stages of life cycle: larvae, pupae, and adults. Shown are percentages of animals that hatched into larvae, pupated into pupae, and eclosed into adults for each genotype. The wildtype shown is for *Ago1^T908M^*/+ heterozygotes. Other wildtype genotypes tested (*Ago1^D743N^*/+, *FRT82B/Df*, and *Ago1^Q127X^*/+) show comparable hatching and eclosion frequencies. Various alleles were placed over respective null alleles to exclude the possibility that secondary mutations on each chromosome contributed to the lethal phenotypes. *Dicer-1* and *Pasha* alleles were placed over chromosome deficiencies (*Df*) that uncovered each locus. The number *n* indicates the number of animals analyzed for each genotype.

As mutations in all four genes were homozygous lethal, we determined the developmental stage at which mutant animals succumbed. Zygotic mutant animals were generated from heterozygous parents, so that mutants received a maternal contribution of gene products. These animals were then monitored for life-cycle transitions: embryos hatching into larvae; larvae pupating into pupae; and pupae eclosing into adults. Wildtype heterozygous animals showed robust numbers passing through the monitored life stages (Figure 7 and Table S1). In contrast, mutant animals exhibited lethality at various stages depending upon the gene and allele. *Ago1* nonsense mutants were embryonic lethal (Figure 7 and Table S1), although they showed normal axis patterning (data not shown). Missense *Ago1* alleles exhibited a phenotypic series, with strong to weak severity following: R937C→E808K→T908M→D743N. *Drosha* and *Pasha* mutants were lethal primarily at the pupal stage of life, whereas *Dicer-1* mutants were lethal during larval and pupal stages. The early lethality displayed by *Ago1* might be due to less perdurance of maternally supplied Ago1 mRNA and protein. Alternatively, it might be due to functions for *Ago1* not attributed to the other genes, as was suggested from the eye development experiments.

### RNA decapping is required for miRNA-mediated silencing

**Figure 8:**
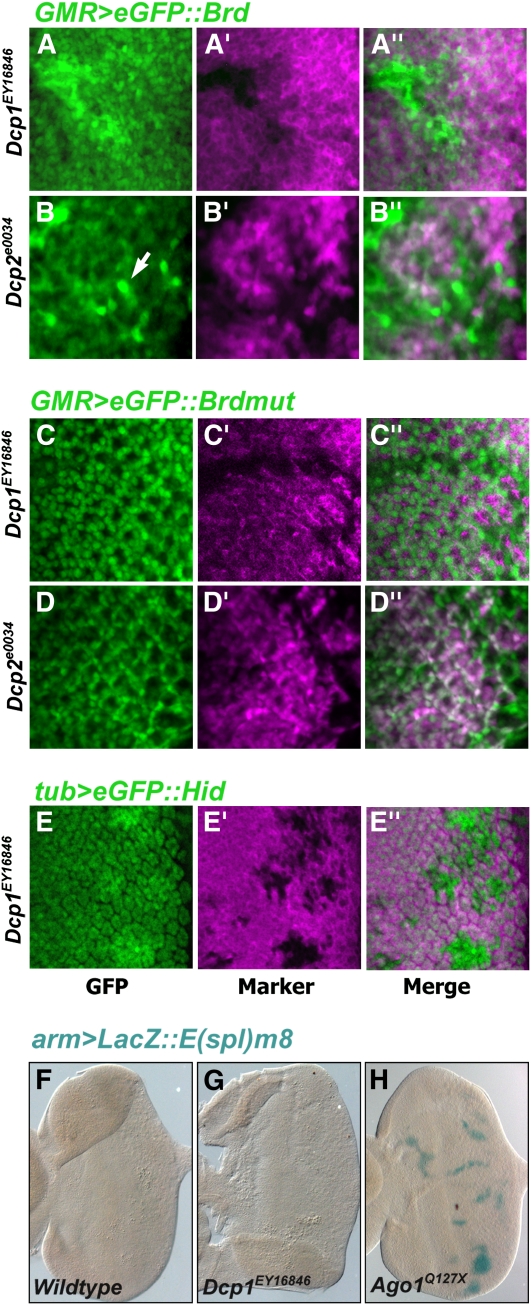
Genes encoding mRNA decapping proteins are required for miRNA silencing. (A–A′′) *GMR > eGFP*::*Brd* expression (green) in *Dcp1* mutant clones marked by the absence of RFP (purple). (B–B′′) *GMR > eGFP*::*Brd* expression (green) in *Dcp2* mutant clones marked by the absence of RFP (purple). Note strong GFP expression in a subset of mutant cells (arrow). We observe that some cells within a clone are strongly derepressed, whereas other cells do not show derepressed sensor expression. The reasons for this are unclear but might have to do with perdurance of Dcp2 in some mutant cells such that repression is maintained longer than in other sibling cells. (C–C′′) *GMR > eGFP*::*Brdmut* expression (green) in *Dcp1* mutant clones marked by the absence of RFP (purple). (D–D′′) *GMR > eGFP*::*Brdmut* expression (green) in *Dcp2* mutant clones marked by the absence of RFP (purple). (E–E′′) *tubulin > eGFP*::*Hid* expression (green) in *Dcp1* mutant clones marked by the absence of LacZ (purple). (F–H) Expression of *arm > LacZ*::*E(spl)m8* in a wildtype eye (F), an eye mosaic for *Dcp1* mutant cells (G), and an eye mosaic for *Ago1* mutant cells (H).

One of the mutants isolated from the screen was an insertional mutation in the *Dcp1* gene. In the developing larval eye, *Dcp1* mutant clones showed strong derepression of *GMR > eGFP*::*Brd*, comparable to levels observed with mutations in the core genes (Figure 8A). Removal of the mRNA 5′ cap structure is catalyzed by the decapping enzyme Dcp2. To be fully active and/or stable, Dcp2 interacts directly with Dcp1, and this interaction is required for decapping *in vivo* and *in vitro* (Coller and Parker 2004). We wished to determine whether Dcp2 is also required for the miRNA repression observed in the *Drosophila* eye. Therefore, we obtained an insertional mutant in the *Drosophila Dcp2* gene, and we examined *GMR > eGFP*::*Brd* reporter expression in marked *Dcp2* mutant clones (Figure 8B). The reporter was derepressed in mutant cells, consistent with the effect of *Dcp1*.

Importantly, the effects of *Dcp1* and *Dcp2* were dependent upon the presence of miRNA binding sites in the reporter 3′UTR; the *GMR > eGFP*::*Brdmut* reporter showed no such derepression within *Dcp1* and *Dcp2* clones (Figure 8, C and D). *Dcp1* clones did not show derepression of the LacZ reporter transgene containing the *E(spl)m8* 3′UTR rather than the *Brd* 3′UTR (Figure 8, F–H). This was a marked discrepancy from the core mutants. One possible explanation is that Dcp1 is highly specific for *Brd* repression. To test this possibility, we also looked at a reporter transgene containing the *Hid* 3′UTR. This UTR contains many miRNA binding sites, including sites for the miRNA *bantam* (Brennecke *et al.* 2003). The *tub > eGFP*::*Hid* reporter showed derepression within *Dcp1* mutant clones (Figure 8E). Thus, mRNA decapping is required *in vivo* to repress some but not all target genes in a miRNA-dependent manner.

## DISCUSSION

Here, we have described a systematic screen for factors important for miRNA silencing in *Drosophila*. Genes within 40% of the genome were identified whose loss could perturb silencing but not lead to cell lethality. Applying Poisson analysis to the screen data, we estimate that 28 to 45 genes whose loss might have been detected are present in that 40% of the genome. A genome-wide RNAi screen for genes in the S2 cell miRNA pathway discovered a total of 98 genes required for silencing (Zhou *et al.* 2008), consistent in magnitude with the results from our animal mutagenesis screen.

We observed that *Ago1* mutants exhibited stronger effects on eye development and embryonic viability than mutants in the other core factors. There are several possible explanations. First, it is possible the mutations in the other genes were not null, but we think this unlikely as multiple nonsense alleles for each gene gave similar phenotypes. Second, *Ago1* might encode the only Argonaute protein to mediate miRNA regulation in *Drosophila*, whereas *Dicer-1*, *Drosha*, and *Pasha* might be redundant with other genes. For example, Dicer-2 can process pre-miRNAs *in vitro* (Cenik *et al.* 2011) and, therefore, could conceivably contribute to some pre-miRNA processing *in vivo*. Mirtrons are processed by the mRNA splicing machinery and not Microprocessor, and therefore, miRNAs made from mirtrons will function in the absence of *Drosha* and *Pasha*. Third, Ago1 might regulate development using small RNAs that are not miRNAs. For example, a small fraction of endo-siRNAs processed by Dicer-2 are associated with Ago1 (Czech *et al.* 2009), and some endo-siRNAs might function in eye development. Finally, Ago1 might process certain miRNAs in a manner independent of the other core factors, as has been found for its mammalian ortholog Ago2 (Cheloufi *et al.* 2010; Cifuentes *et al.* 2010).

The core mutants displayed another surprising character. The mutants caused repression of the *GMR > eGFP*::*Brdmut* sensor, which contrasts with the direct role that the wildtype genes play in gene repression. We do not think that this result uncovers a function for the genes in directly activating expression. Rather, it is likely that the mutants derepress the expression of a transcription or translation factor, which in turn inhibits the expression of the sensor.

Three candidates emerging from the screen were chromatin-modifying factors. Grappa is the Dot1 ortholog, a histone H3 K79 methyltransferase that is required for chromatin silencing (Shanower *et al.* 2005). Interestingly, Grappa was also identified from the genome-wide S2 cell screen as a factor weakly required for miRNA-mediated silencing (Zhou *et al.* 2008). Altogether, it would suggest that Grappa regulates diverse RNA-dependent repression in a variety of *Drosophila* cell types. A second factor, Bap55, physically associates with the Brahma chromatin-remodeling complex in *Drosophila* embryo extracts (Armstrong *et al.* 2002). Bap55 also associates with Domino (a DNA-dependent ATPase) to form the TIP60 histone acetyltransferase complex (Kusch *et al.* 2004). TIP60 has been shown to be involved in many processes, including both transcriptional activation and repression (Sapountzi *et al.* 2006). As we identified both *Bap55* and *Domino* from our genetic screen, it would suggest that both factors function in miRNA silencing as part of the TIP60 complex, although we cannot yet exclude the possibility that other remodeling complexes might also play a role. Zhou *et al.* (2008) also identified *Domino* from their RNAi-based screen, although RNAi of *Domino* led to stronger repression than normal. *Bap55* was not identified from their screen. Interestingly, the TIP60 complex has been implicated in dendrite patterning of olfactory projection neurons of *Drosophila* (Tea and Luo 2011), a wiring process that is also regulated by the miRNA pathway (Berdnik *et al.* 2008).

### Genetic evidence that decapping is important for miRNA-mediated repression

We identified the mRNA decapping genes *Dcp1* and *Dcp2* as factors required for silencing some but not all miRNA targets. This is the first genetic evidence in animals that decapping is required for miRNA-mediated repression. Earlier studies relying upon S2 cell RNAi depletion and biochemistry had implicated decapping enzymes as destabilizers of miRNA-targeted transcripts (Behm-Ansmant *et al.* 2006; Eulalio *et al.* 2007). Moreover, miRNA targets are frequently localized to P-bodies, cytoplasmic foci rich in decapping proteins (Liu *et al.* 2005b). We found that *Dcp1* is not required to silence all reporters we tested. This is consistent with S2 cell knockdowns in which miRNA targets displayed differential sensitivity to loss of decapping (Eulalio *et al.* 2007). Targets that were repressed primarily through translation and not mRNA decay were less sensitive to loss of decapping. It was suggested that targets with shorter 3′UTRs (<500 nts) are less sensitive to decapping enzymes. However, the *E(spl)m8* gene 3′UTR is much longer than the 213 nt *Brd* 3′UTR, yet *E(spl)m8* is insensitive to *Dcp1* whereas *Brd* is quite responsive. The precise features of these miRNA targets that make silencing dependent upon decapping are unknown.

### A collection of null mutations in core genes

We have provided a rich genetic resource for the further study of the miRNA pathway of *Drosophila*. Null mutations have been isolated in *Drosha*, *Pasha*, *Dicer-1*, and *Ago1*. The latter gene in particular has been problematic in terms of generating null mutations. A recent EMS screen failed to detect any *Ago1* mutations (Smibert *et al.* 2011). There are four *Ago1* transcripts: isoforms C and D, with a common promoter, and isoforms A and B, each with unique promoters. *P*-element insertional mutations have been described that are located either in the second intron of C and D transcripts or in the 5′UTR of the B transcript (Grimaud *et al.* 2006; Kataoka *et al.* 2001; Roch *et al.* 1998). These mutations appear hypomorphic in nature. The EMS mutations in Ago1 that we recovered are predicted to disrupt the coding sequence of all four transcripts. Some of these mutations are protein-null and will therefore provide useful genetic tools for the *Drosophila* community.

## Supplementary Material

Supporting Information

## References

[bib1] Aleman L. M. Doench J. Sharp P. A., 2007 Comparison of siRNA-induced off-target RNA and protein effects. RNA 13: 385–395 17237357 10.1261/rna.352507 PMC1800510

[bib2] Armstrong J. A. Papoulas O. Daubresse G. Sperling A. S. Lis J. T., 2002 The Drosophila BRM complex facilitates global transcription by RNA polymerase II. EMBO J. 21: 5245–5254 12356740 10.1093/emboj/cdf517 PMC129039

[bib3] Bartel D. P., 2009 MicroRNAs: target recognition and regulatory functions. Cell 136: 215–233 19167326 10.1016/j.cell.2009.01.002 PMC3794896

[bib4] Behm-Ansmant I. Rehwinkel J. Doerks T. Stark A. Bork P., 2006 mRNA degradation by miRNAs and GW182 requires both CCR4:NOT deadenylase and DCP1:DCP2 decapping complexes. Genes Dev. 20: 1885–1898 16815998 10.1101/gad.1424106 PMC1522082

[bib5] Berdnik D. Fan A. P. Potter C. J. Luo L., 2008 MicroRNA processing pathway regulates olfactory neuron morphogenesis. Curr. Biol. 18: 1754–1759 19013069 10.1016/j.cub.2008.09.045 PMC2612040

[bib6] Boland A. Huntzinger E. Schmidt S. Izaurralde E. Weichenrieder O., 2011 Crystal structure of the MID-PIWI lobe of a eukaryotic Argonaute protein. Proc. Natl. Acad. Sci. USA 108: 10466–10471 21646546 10.1073/pnas.1103946108 PMC3127882

[bib7] Brennecke J. Hipfner D. R. Stark A. Russell R. B. Cohen S. M., 2003 bantam encodes a developmentally regulated microRNA that controls cell proliferation and regulates the proapoptotic gene hid in Drosophila. Cell 113: 25–36 12679032 10.1016/s0092-8674(03)00231-9

[bib8] Bushati N. Cohen S. M., 2007 microRNA functions. Annu. Rev. Cell Dev. Biol. 23: 175–205 17506695 10.1146/annurev.cellbio.23.090506.123406

[bib9] Carthew R. W. Sontheimer E. J., 2009 Origins and Mechanisms of miRNAs and siRNAs. Cell 136: 642–655 19239886 10.1016/j.cell.2009.01.035 PMC2675692

[bib10] Cenik E. S. Fukunaga R. Lu G. Dutcher R. Wang Y., 2011 Phosphate and R2D2 restrict the substrate specificity of Dicer-2, an ATP-driven ribonuclease. Mol. Cell 42: 172–184 21419681 10.1016/j.molcel.2011.03.002 PMC3115569

[bib11] Cheloufi S. Dos Santos C. O. Chong M. M. Hannon G. J., 2010 A dicer-independent miRNA biogenesis pathway that requires Ago catalysis. Nature 465: 584–589 20424607 10.1038/nature09092 PMC2995450

[bib12] Chen J. Call G. B. Beyer E. Bui C. Cespedes A., 2005 Discovery-based science education: functional genomic dissection in Drosophila by undergraduate researchers. PLoS Biol. 3: e59 15719063 10.1371/journal.pbio.0030059 PMC548953

[bib13] Cifuentes D. Xue H. Taylor D. W. Patnode H. Mishima Y., 2010 A novel miRNA processing pathway independent of Dicer requires Argonaute2 catalytic activity. Science 328: 1694–1698 20448148 10.1126/science.1190809 PMC3093307

[bib14] Coller J. Parker R., 2004 Eukaryotic mRNA decapping. Annu. Rev. Biochem. 73: 861–890 15189161 10.1146/annurev.biochem.73.011303.074032

[bib15] Couchman J. R., 2003 Syndecans: proteoglycan regulators of cell-surface microdomains? Nat. Rev. Mol. Cell Biol. 4: 926–937 14685171 10.1038/nrm1257

[bib16] Czech B. Hannon G. J., 2011 Small RNA sorting: matchmaking for Argonautes. Nat. Rev. Genet. 12: 19–31 21116305 10.1038/nrg2916 PMC3703915

[bib17] Czech B. Zhou R. Erlich Y. Brennecke J. Binari R., 2009 Hierarchical rules for Argonaute loading in Drosophila. Mol. Cell 36: 445–456 19917252 10.1016/j.molcel.2009.09.028 PMC2795325

[bib18] Denli A. M. Tops B. B. Plasterk R. H. Ketting R. F. Hannon G. J., 2004 Processing of primary microRNAs by the Microprocessor complex. Nature 432: 231–235 15531879 10.1038/nature03049

[bib19] Eulalio A. Rehwinkel J. Stricker M. Huntzinger E. Yang S. F., 2007 Target-specific requirements for enhancers of decapping in miRNA-mediated gene silencing. Genes Dev. 21: 2558–2570 17901217 10.1101/gad.443107 PMC2000321

[bib20] Eulalio A. Huntzinger E. Izaurralde E., 2008 GW182 interaction with Argonaute is essential for miRNA-mediated translational repression and mRNA decay. Nat. Struct. Mol. Biol. 15: 346–353 18345015 10.1038/nsmb.1405

[bib21] Filipowicz W. Bhattacharyya S. N. Sonenberg N., 2008 Mechanisms of post-transcriptional regulation by microRNAs: are the answers in sight? Nat. Rev. Genet. 9: 102–114 18197166 10.1038/nrg2290

[bib22] Forstemann K. Tomari Y. Du T. Vagin V. V. Denli A. M., 2005 Normal microRNA maturation and germ-line stem cell maintenance requires Loquacious, a double-stranded RNA-binding domain protein. PLoS Biol. 3: e236 15918770 10.1371/journal.pbio.0030236 PMC1141267

[bib23] Gloor G. B. Preston C. R. Johnson-Schlitz D. M. Nassif N. A. Phillis R. W., 1993 Type I repressors of P element mobility. Genetics 135: 81–95 8224830 10.1093/genetics/135.1.81 PMC1205629

[bib24] Gregory R. I. Yan K. P. Amuthan G. Chendrimada T. Doratotaj B., 2004 The Microprocessor complex mediates the genesis of microRNAs. Nature 432: 235–240 15531877 10.1038/nature03120

[bib25] Grimaud C. Bantignies F. Pal-Bhadra M. Ghana P. Bhadra U., 2006 RNAi components are required for nuclear clustering of Polycomb group response elements. Cell 124: 957–971 16530043 10.1016/j.cell.2006.01.036

[bib26] Huntzinger E. Izaurralde E., 2011 Gene silencing by microRNAs: contributions of translational repression and mRNA decay. Nat. Rev. Genet. 12: 99–110 21245828 10.1038/nrg2936

[bib27] Jenkins J. B., 1967 Mutagenesis at a complex locus in *Drosophila* with the monofunctional alkylating agent, ethyl methanesulfonate. Genetics 57: 783–793 6082622 10.1093/genetics/57.4.783 PMC1211767

[bib28] Karim F. D. Chang H. C. Therrien M. Wassarman D. A. Laverty T., 1996 A screen for genes that function downstream of Ras1 during *Drosophila* eye development. Genetics 143: 315–329 8722784 10.1093/genetics/143.1.315 PMC1207264

[bib29] Kataoka Y. Takeichi M. Uemura T., 2001 Developmental roles and molecular characterization of a Drosophila homologue of Arabidopsis Argonaute1, the founder of a novel gene superfamily. Genes Cells 6: 313–325 11318874 10.1046/j.1365-2443.2001.00427.x

[bib30] Kim V. N., 2005 MicroRNA biogenesis: coordinated cropping and dicing. Nat. Rev. Mol. Cell Biol. 6: 376–385 15852042 10.1038/nrm1644

[bib31] Kusch T. Florens L. Macdonald W. H. Swanson S. K. Glaser R. L., 2004 Acetylation by Tip60 is required for selective histone variant exchange at DNA lesions. Science 306: 2084–2087 15528408 10.1126/science.1103455

[bib32] Lai E. C. Posakony J. W., 1997 The Bearded box, a novel 3′ UTR sequence motif, mediates negative post-transcriptional regulation of Bearded and Enhancer of split Complex gene expression. Development 124: 4847–4856 9428421 10.1242/dev.124.23.4847

[bib33] Lai E. C. Tam B. Rubin G. M., 2005 Pervasive regulation of Drosophila Notch target genes by GY-box-, Brd-box-, and K-box-class microRNAs. Genes Dev. 19: 1067–1080 15833912 10.1101/gad.1291905 PMC1091741

[bib34] Le T. Liang Z. Patel H. Yu M. H. Sivasubramaniam G., 2006 A new family of *Drosophila* balancer chromosomes with a w-dfd-GMR yellow fluorescent protein marker. Genetics 174: 2255–2257 17057238 10.1534/genetics.106.063461 PMC1698648

[bib35] Lee Y. Ahn C. Han J. Choi H. Kim J., 2003 The nuclear RNase III Drosha initiates microRNA processing. Nature 425: 415–419 14508493 10.1038/nature01957

[bib36] Lee Y. S. Nakahara K. Pham J. W. Kim K. He Z., 2004 Distinct roles for Drosophila Dicer-1 and Dicer-2 in the siRNA/miRNA silencing pathways. Cell 117: 69–81 15066283 10.1016/s0092-8674(04)00261-2

[bib37] Lewis E. B. Bacher F., 1968 Method of feeding ethyl methanesulfonate to Drosophila males. Drosoph. Inf. Serv. 43: 193–194

[bib38] Li X. Carthew R. W., 2005 A microRNA mediates EGF receptor signaling and promotes photoreceptor differentiation in the Drosophila eye. Cell 123: 1267–1277 16377567 10.1016/j.cell.2005.10.040

[bib39] Liu J. Rivas F. V. Wohlschlegel J. Yates J. R. 3rd Parker R., 2005 a A role for the P-body component GW182 in microRNA function. Nat. Cell Biol. 7: 1261–1266 16284623 10.1038/ncb1333 PMC1804202

[bib40] Liu J. Valencia-Sanchez M. A. Hannon G. J. Parker R., 2005 b MicroRNA-dependent localization of targeted mRNAs to mammalian P-bodies. Nat. Cell Biol. 7: 719–723 15937477 10.1038/ncb1274 PMC1855297

[bib41] Macrae I. J. Zhou K. Li F. Repic A. Brooks A. N., 2006 Structural basis for double-stranded RNA processing by Dicer. Science 311: 195–198 16410517 10.1126/science.1121638

[bib42] Martin R. Smibert P. Yalcin A. Tyler D. M. Schafer U., 2009 A Drosophila pasha mutant distinguishes the canonical microRNA and mirtron pathways. Mol. Cell. Biol. 29: 861–870 19047376 10.1128/MCB.01524-08 PMC2630677

[bib43] Meister G. Landthaler M. Peters L. Chen P. Y. Urlaub H., 2005 Identification of novel argonaute-associated proteins. Curr. Biol. 15: 2149–2155 16289642 10.1016/j.cub.2005.10.048

[bib44] Miyoshi K. Tsukumo H. Nagami T. Siomi H. Siomi M. C., 2005 Slicer function of Drosophila Argonautes and its involvement in RISC formation. Genes Dev. 19: 2837–2848 16287716 10.1101/gad.1370605 PMC1315391

[bib45] Newsome T. P. Asling B. Dickson B. J., 2000 Analysis of Drosophila photoreceptor axon guidance in eye-specific mosaics. Development 127: 851–860 10648243 10.1242/dev.127.4.851

[bib46] Nüsslein-Volhard C. Wieschaus E. Kluding H., 1984 Mutations affecting the pattern of the larval cuticle in Drosophila melanogaster I. Zygotic loci on the second chromosome. Dev. Genes Evol. 193: 267–282 10.1007/BF00848156 28305337

[bib47] Parker J. S. Roe S. M. Barford D., 2004 Crystal structure of a PIWI protein suggests mechanisms for siRNA recognition and slicer activity. EMBO J. 23: 4727–4737 15565169 10.1038/sj.emboj.7600488 PMC535097

[bib48] Parry D. H. Xu J. Ruvkun G., 2007 A whole-genome RNAi screen for C. elegans miRNA pathway genes. Curr. Biol. 17: 2013–2022 18023351 10.1016/j.cub.2007.10.058 PMC2211719

[bib49] Rivas F. V. Tolia N. H. Song J. J. Aragon J. P. Liu J., 2005 Purified Argonaute2 and an siRNA form recombinant human RISC. Nat. Struct. Mol. Biol. 12: 340–349 15800637 10.1038/nsmb918

[bib50] Roch F. Serras F. Cifuentes F. J. Corominas M. Alsina B., 1998 Screening of larval/pupal P-element induced lethals on the second chromosome in Drosophila melanogaster: clonal analysis and morphology of imaginal discs. Mol. Gen. Genet. 257: 103–112 9491068 10.1007/pl00008620

[bib51] Ruby J. G. Jan C. H. Bartel D. P., 2007 Intronic microRNA precursors that bypass Drosha processing. Nature 448: 83–86 17589500 10.1038/nature05983 PMC2475599

[bib52] Saito K. Ishizuka A. Siomi H. Siomi M. C., 2005 Processing of pre-microRNAs by the Dicer-1-Loquacious complex in Drosophila cells. PLoS Biol. 3: e235 15918769 10.1371/journal.pbio.0030235 PMC1141268

[bib53] Sapountzi V. Logan I. R. Robson C. N., 2006 Cellular functions of TIP60. Int. J. Biochem. Cell Biol. 38: 1496–1509 16698308 10.1016/j.biocel.2006.03.003

[bib54] Schuldiner O. Berdnik D. Levy J. M. Wu J. S. Luginbuhl D., 2008 piggyBac-based mosaic screen identifies a postmitotic function for cohesin in regulating developmental axon pruning. Dev. Cell 14: 227–238 18267091 10.1016/j.devcel.2007.11.001 PMC2268086

[bib55] Shanower G. A. Muller M. Blanton J. L. Honti V. Gyurkovics H., 2005 Characterization of the grappa gene, the *Drosophila* histone H3 lysine 79 methyltransferase. Genetics 169: 173–184 15371351 10.1534/genetics.104.033191 PMC1448877

[bib56] Smibert P. Bejarano F. Wang D. Garaulet D. L. Yang J. S., 2011 A Drosophila genetic screen yields allelic series of core microRNA biogenesis factors and reveals post-developmental roles for microRNAs. RNA 11: 1997–2010 21947201 10.1261/rna.2983511 PMC3198593

[bib57] Song J. J. Smith S. K. Hannon G. J. Joshua-Tor L., 2004 Crystal structure of Argonaute and its implications for RISC slicer activity. Science 305: 1434–1437 15284453 10.1126/science.1102514

[bib58] Spradling A. C. Stern D. Beaton A. Rhem E. J. Laverty T., 1999 The Berkeley Drosophila Genome Project gene disruption project: single P-element insertions mutating 25% of vital *Drosophila* genes. Genetics 153: 135–177 10471706 10.1093/genetics/153.1.135 PMC1460730

[bib59] Spring J. Paine-Saunders S. E. Hynes R. O. Bernfield M., 1994 Drosophila syndecan: conservation of a cell-surface heparan sulfate proteoglycan. Proc. Natl. Acad. Sci. USA 91: 3334–3338 8159748 10.1073/pnas.91.8.3334 PMC43571

[bib60] Stowers R. S. Schwarz T. L., 1999 A genetic method for generating *Drosophila* eyes composed exclusively of mitotic clones of a single genotype. Genetics 152: 1631–1639 10430588 10.1093/genetics/152.4.1631 PMC1460682

[bib61] Tea J. S. Luo L., 2011 The chromatin remodeling factor Bap55 functions through the TIP60 complex to regulate olfactory projection neuron dendrite targeting. Neural Dev. 6: 5 21284845 10.1186/1749-8104-6-5 PMC3038883

[bib62] Till S. Lejeune E. Thermann R. Bortfeld M. Hothorn M., 2007 A conserved motif in Argonaute-interacting proteins mediates functional interactions through the Argonaute PIWI domain. Nat. Struct. Mol. Biol. 14: 897–903 17891150 10.1038/nsmb1302

[bib63] Xu C. Kauffmann R. C. Zhang J. Kladny S. Carthew R. W., 2000 Overlapping activators and repressors delimit transcriptional response to receptor tyrosine kinase signals in the Drosophila eye. Cell 103: 87–97 11051550 10.1016/s0092-8674(00)00107-0

[bib64] Xu T. Rubin G. M., 1993 Analysis of genetic mosaics in developing and adult Drosophila tissues. Development 117: 1223–1237 8404527 10.1242/dev.117.4.1223

[bib65] Yang W. Steitz T. A., 1995 Recombining the structures of HIV integrase, RuvC and RNase H. Structure 3: 131–134 7735828 10.1016/s0969-2126(01)00142-3

[bib66] Zhai R. G. Hiesinger P. R. Koh T. W. Verstreken P. Schulze K. L., 2003 Mapping Drosophila mutations with molecularly defined P element insertions. Proc. Natl. Acad. Sci. USA 100: 10860–10865 12960394 10.1073/pnas.1832753100 PMC196893

[bib67] Zhou R. Hotta I. Denli A. M. Hong P. Perrimon N., 2008 Comparative analysis of argonaute-dependent small RNA pathways in Drosophila. Mol. Cell 32: 592–599 19026789 10.1016/j.molcel.2008.10.018 PMC2615197

