# Supplementary material for: A Systematic Genetic Screen to Dissect the MicroRNA Pathway in Drosophila
Source: G3 (Bethesda). 2012 Apr 1;2(4):437–48. doi: 10.1534/g3.112.002030 (PMC3337472; doi:10.1534/g3.112.002030)
Supplement: Supporting Information [file supp_2_4_437__index.html]

Supporting Information 

# A Systematic Genetic Screen to Dissect the MicroRNA Pathway in *Drosophila*

## Supporting Information for Pressman *et al.*, 2012

**Files in this Data Supplement:**

- Supporting Information - Figures S1-S10 and Table S1 (PDF, 1.1 MB)
- Figure S1 - Mutagenesis and crossing scheme for isolation of mutations on right arm of chromosome 2 (PDF, 101 KB)
- Figure S2 - Schematic of Ago1, Dicer-1, Drosha, and Pasha polypeptides showing the conserved domains present in each protein (PDF, 115 KB)
- Figure S3 - Ago1 protein (purple) stained with a monoclonal antibody in a larval eye disc containing clones of Ago1*Q127X* mutant cells (PDF, 109 KB)
- Figure S4 - Alignment of Piwi domains from *Drosophila melanogaster* Ago1 and Ago2, *Xenopus laevis* Ago2, *Mus musculus* Ago2, archaebacterial *Pyrococcus fuiosus* Ago, and eubacterial *Thermus thermophilis* Ago (PDF, 265 KB)
- Figure S5 - Expression of protein from *GMR>eGFP::Brd* (green) in mosaic larval eye discs containing clones of mutant cells homozygous for *Ago1* alleles *J04* (A), *Q127X* (B), *W894X* (C), and *R839X* (D) (PDF, 142 KB)
- Figure S6 - Expression of protein from *GMR>eGFP::Brd* (green) in mosaic larval eye discs containing clones of mutant cells homozygous for *Ago1* missense alleles *T908M* (A), *D743N* (B), *R937C* (C), and *E808K* (D) (PDF, 163 KB)
- Figure S7 - Expression of protein from *GMR>eGFP::Brd* (green) in mosaic larval eye discs containing clones of mutant cells homozygous for *Drosha* alleles *W1123X* (A), *Q884X* (B), and *Q938X* (C) (PDF, 128 KB)
- Figure S8 - Expression of protein from *GMR>eGFP::Brd* (green) in mosaic larval eye discs containing clones of mutant cells homozygous for *Dicer-1* alleles *Q1233X* (A), *Q770X* (B), *W94X* (C), *Q991X* (D), *Q1712X* (E), *Q396X* (F), *K43X* (G), and *G2035S* (H) (PDF, 175 KB)
- Figure S9 - Expression of protein from *GMR>eGFP::Brd* (green) in mosaic larval eye discs containing clones of mutant cells homozygous for *Pasha* alleles *Q394X* (A), *Q83X* (B), *Q579X* (C), *P203L* (D), *A13* (E), and *R59X* (F) (PDF, 189 KB)
- Figure S10 - Expression of protein from *tub>eGFP::2x(miR-7)* (green) where two perfect binding sites for miR-7 are positioned in the 3'UTR (PDF, 176 KB)
- Table S1 - Lethal Phase Analysis of Zygotic Mutants (PDF, 73 KB)
