# Supplementary material for: A Systematic Genetic Screen to Dissect the MicroRNA Pathway in Drosophila
Source: G3 (Bethesda). 2012 Apr 1;2(4):437–48. doi: 10.1534/g3.112.002030 (PMC3337472; doi:10.1534/g3.112.002030)
Supplement: Supporting Information [file supp_2.4.437_002030SI.pdf]

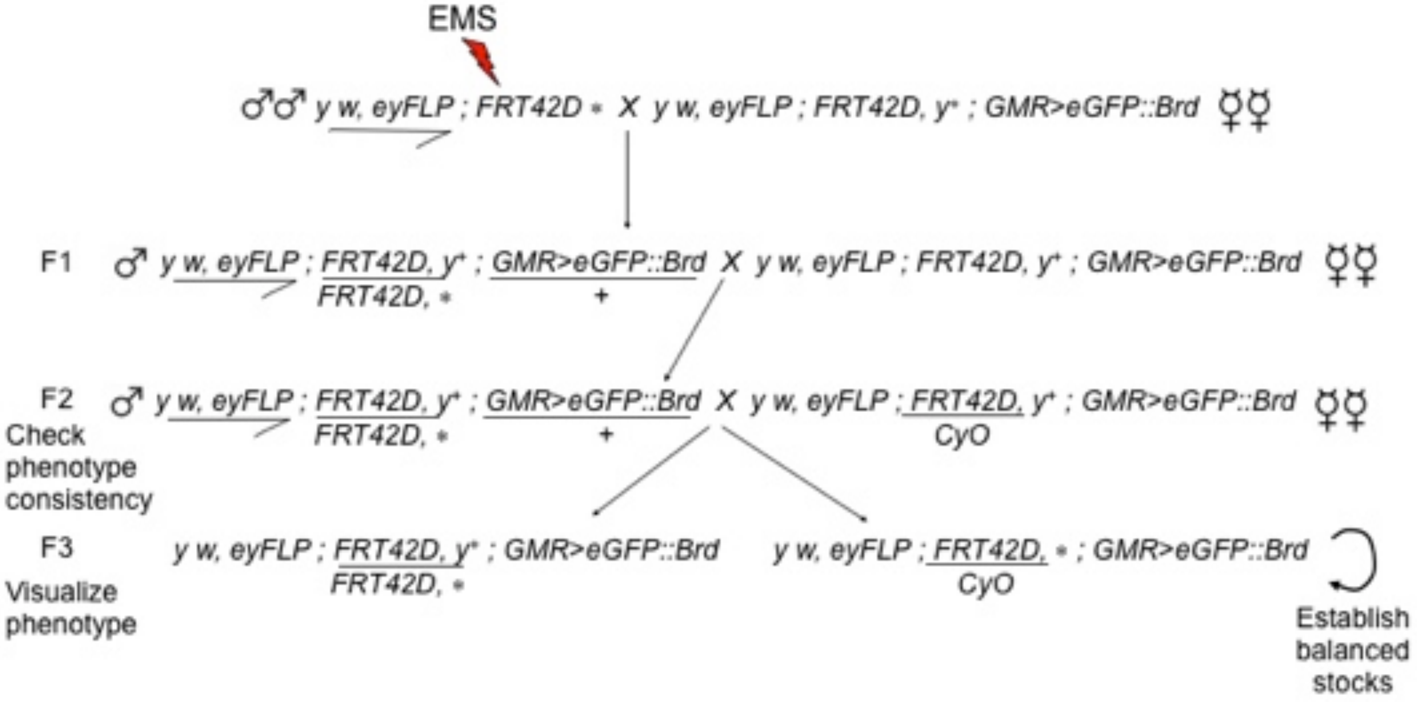

Figure S1. Mutagenesis and crossing scheme for isolation of mutations on right arm of chromosome 2.

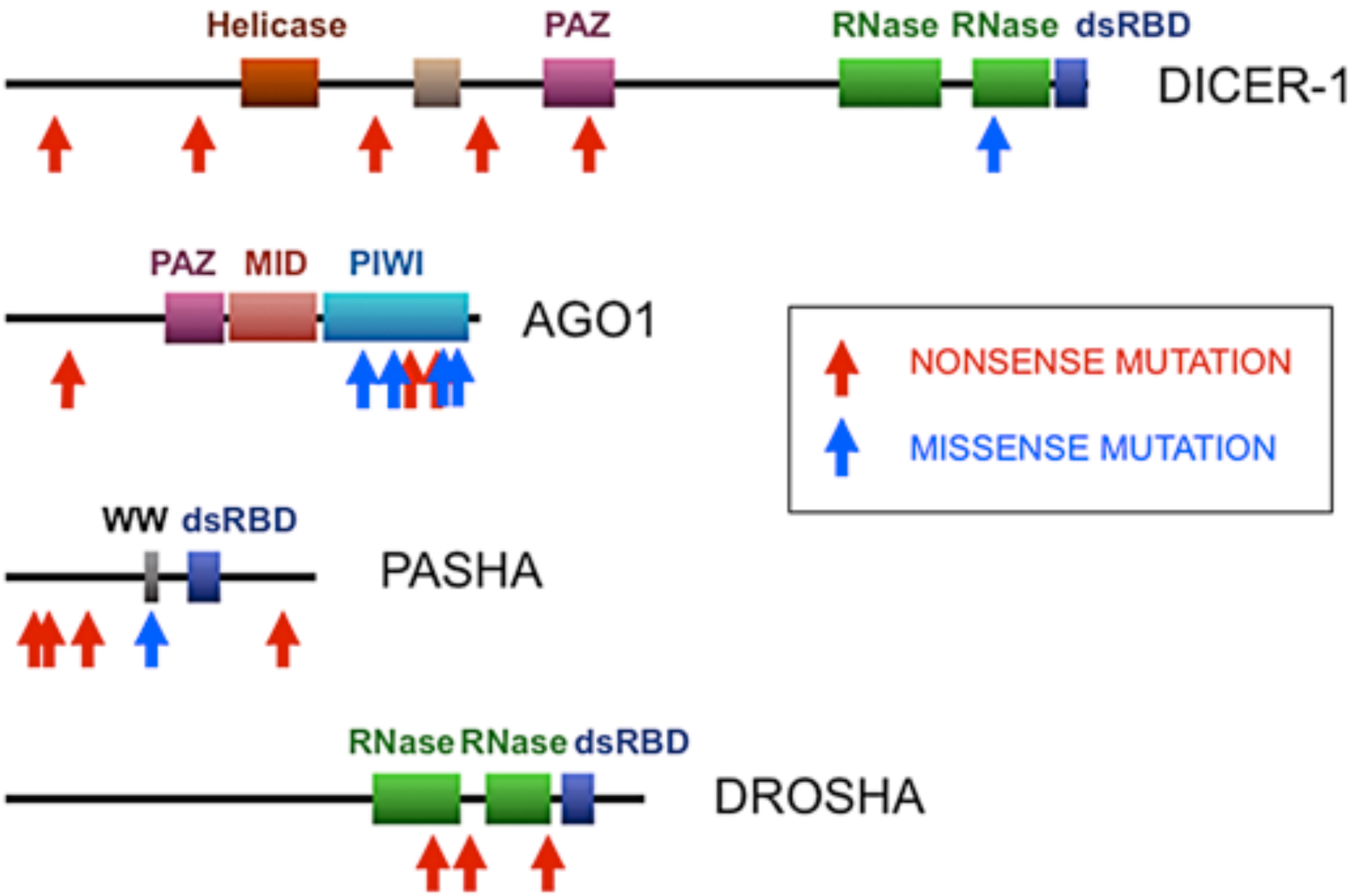

Figure S2. Schematic of Ago1, Dicer-1, Drosha, and Pasha polypeptides showing the conserved domains present in each protein. Indicated are the positions and of the various point mutations for each mutant allele that were isolated in the screen.

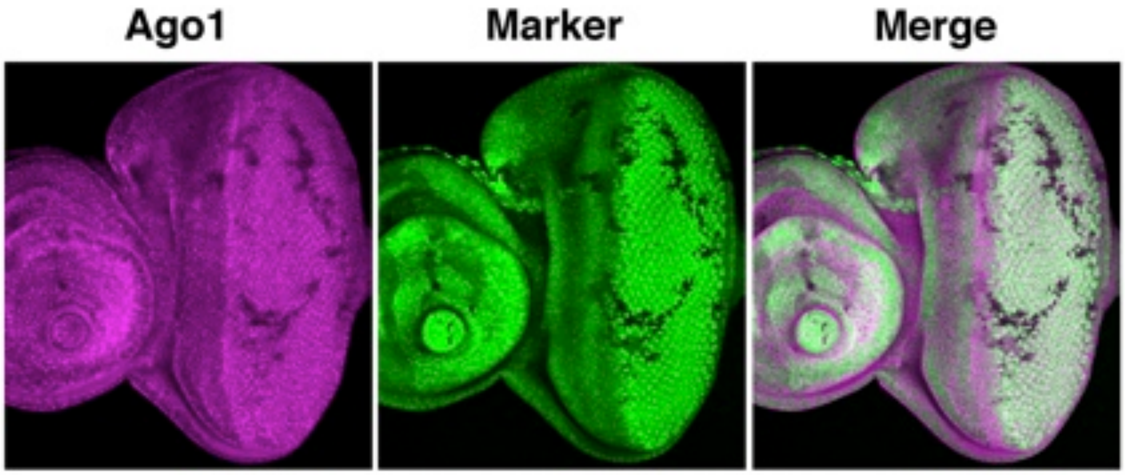

Figure S3. Ago1 protein (purple) stained with a monoclonal antibody in a larval eye disc containing clones of *Ago1*^{Q127X} mutant cells. These mutant cells are marked by the absence of a GFP marker (green); all GFP-positive cells contain one or two copies of the wildtype *Ago1* allele.

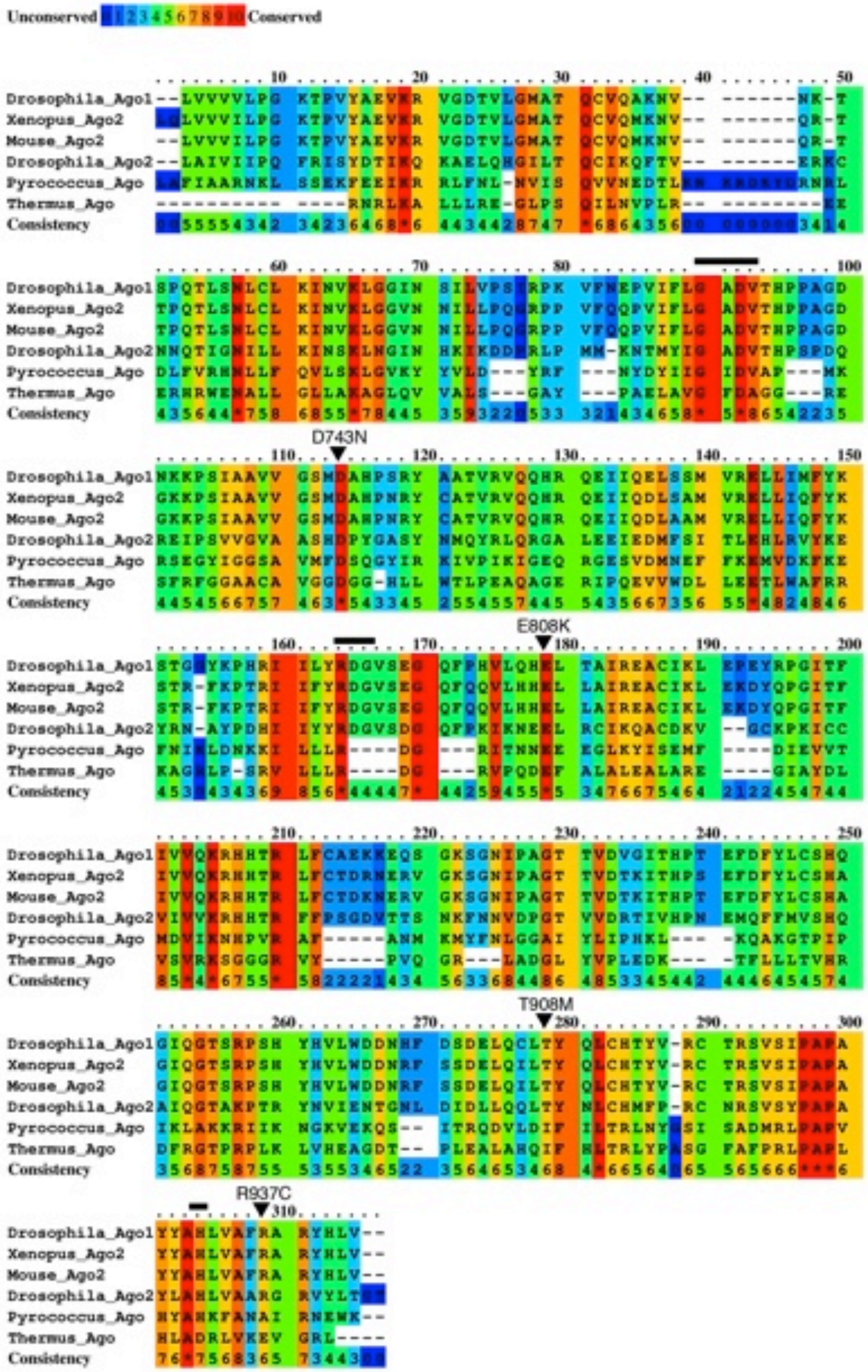

Figure S4. Alignment of Piwi domains from *Drosophila melanogaster* Ago1 and Ago2, *Xenopus laevis* Ago2, *Mus musculus* Ago2, archaebacterial *Pyrococcus fuiosus* Ago, and eubacterial *Thermus thermophilis* Ago. Color coding denotes degree of sequence conservation as indicated at top. Positions of the three clusters that coordinate the metal ion for RNA catalysis are indicated by black bars. Positions of the four residues mutated in missense Ago1 alleles are indicated by black triangles. Alignment was performed using the PRALINE algorithm.

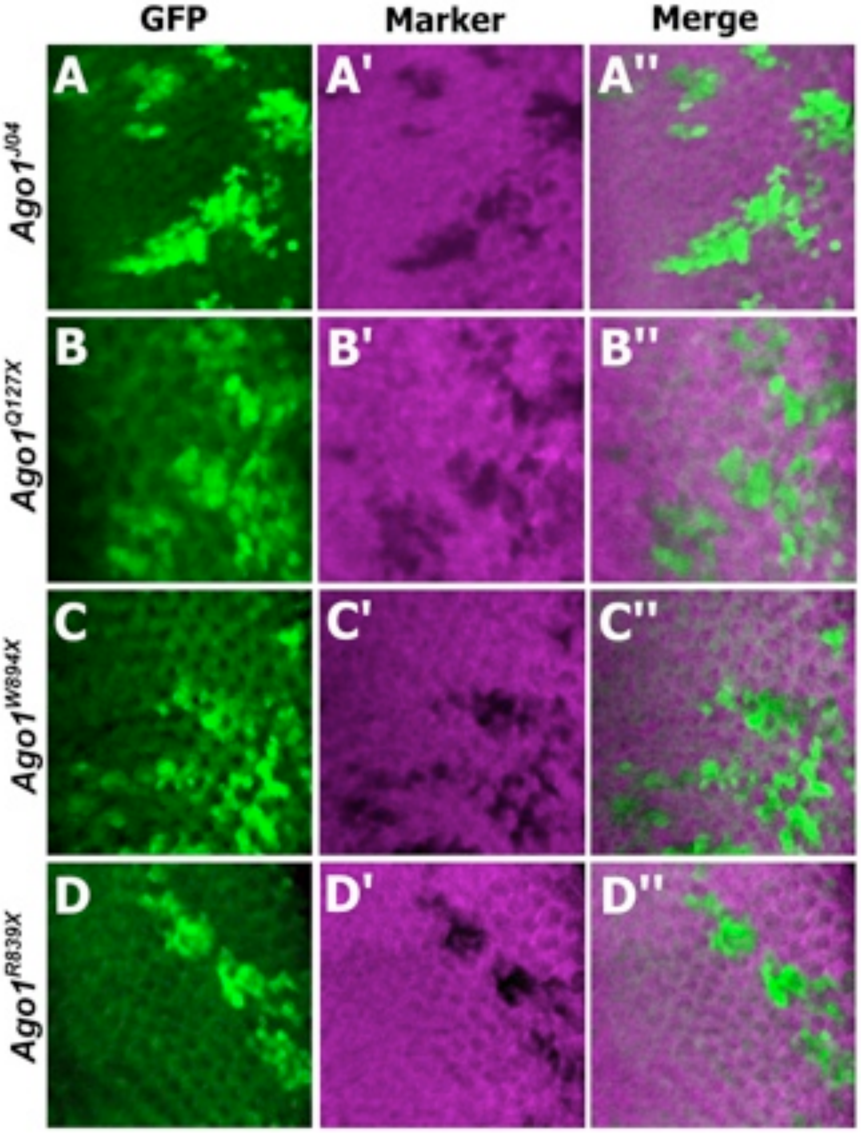

Figure S5. Expression of protein from *GMR>eGFP::Brd* (green) in mosaic larval eye discs containing clones of mutant cells homozygous for *Ago1* alleles *J04* (A), *Q127X* (B), *W894X* (C), and *R839X* (D). Mutant cells are marked by the absence of LacZ protein (purple); cells with one or two copies of the wildtype *Ago1* allele express LacZ.

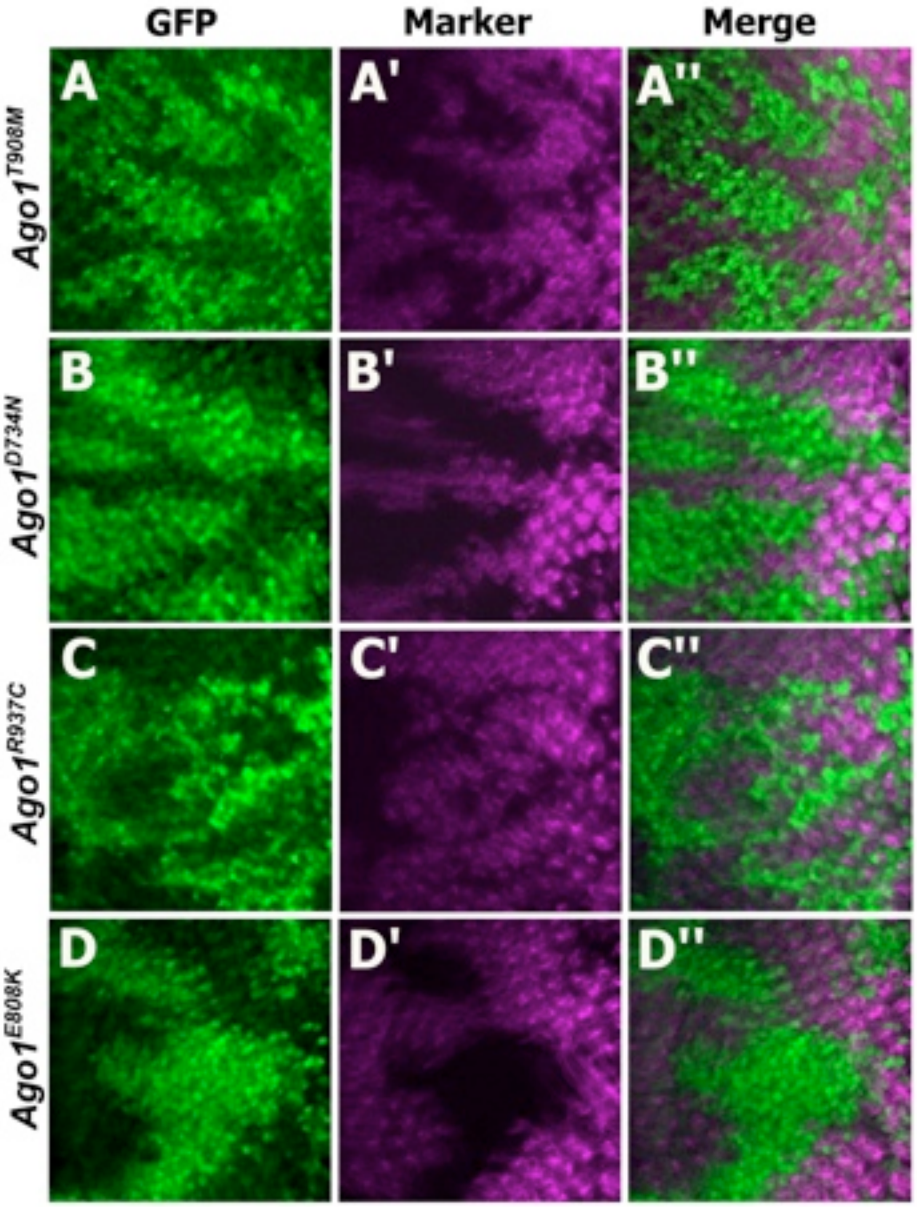

Figure S6. Expression of protein from *GMR>eGFP::Brd* (green) in mosaic larval eye discs containing clones of mutant cells homozygous for *Ago1* missense alleles *T908M* (A), *D743N* (B), *R937C* (C), and *E808K* (D). Mutant cells are marked by the absence of RFP protein (purple); cells with one or two copies of the wildtype *Ago1* allele express RFP.

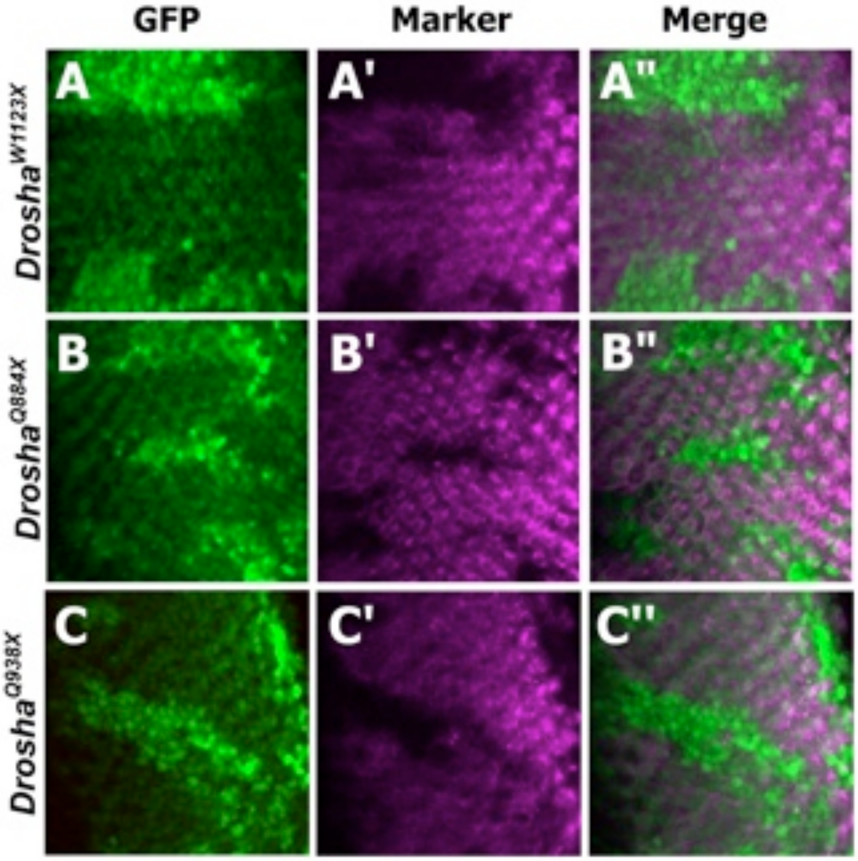

Figure S7. Expression of protein from *GMR>eGFP::Brd* (green) in mosaic larval eye discs containing clones of mutant cells homozygous for *Drosha* alleles *W1123X* (A), *Q884X* (B), and *Q938X* (C). Mutant cells are marked by the absence of RFP protein (purple); cells with one or two copies of the wildtype *Drosha* allele express RFP.

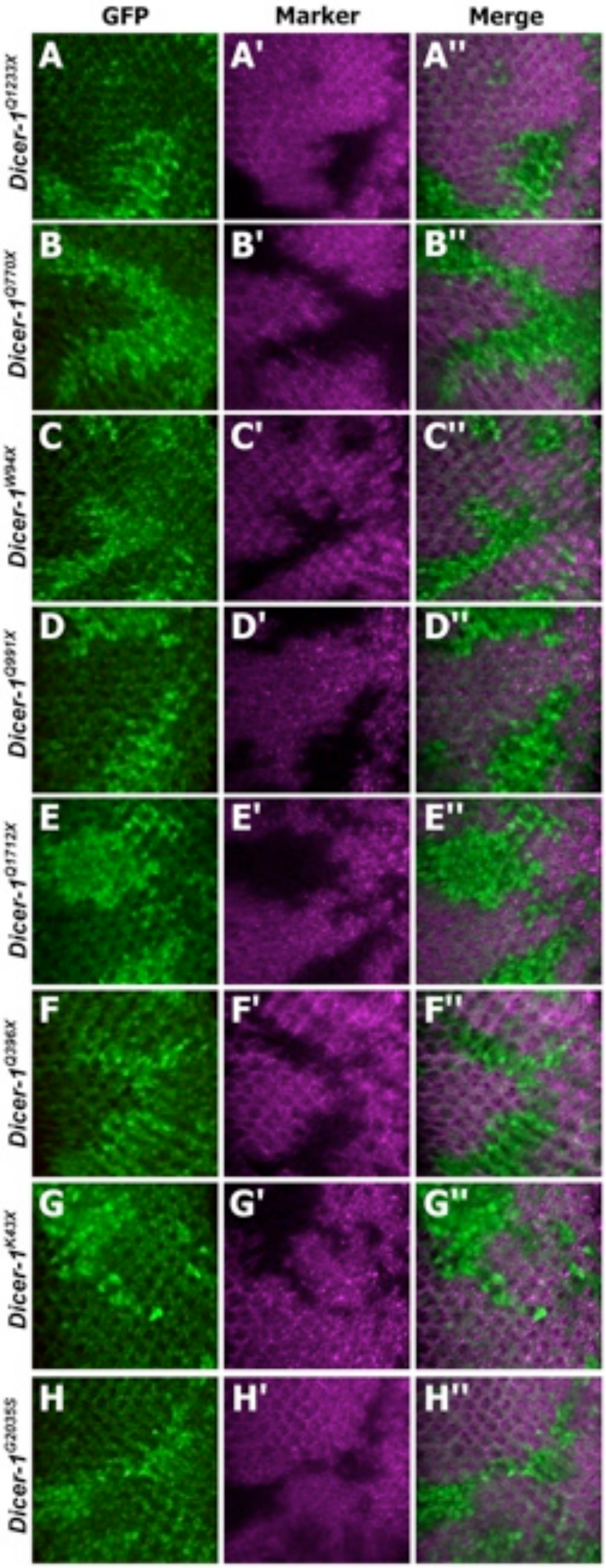

Figure S8. Expression of protein from *GMR>eGFP::Brd* (green) in mosaic larval eye discs containing clones of mutant cells homozygous for *Dicer-1* alleles *Q1233X* (A), *Q770X* (B), *W94X* (C), *Q991X* (D), *Q1712X* (E), *Q396X* (F), *K43X* (G), and *G2035S* (H). Mutant cells are marked by the absence of RFP protein (purple); cells with one or two copies of the wildtype *Dicer-1* allele express RFP.

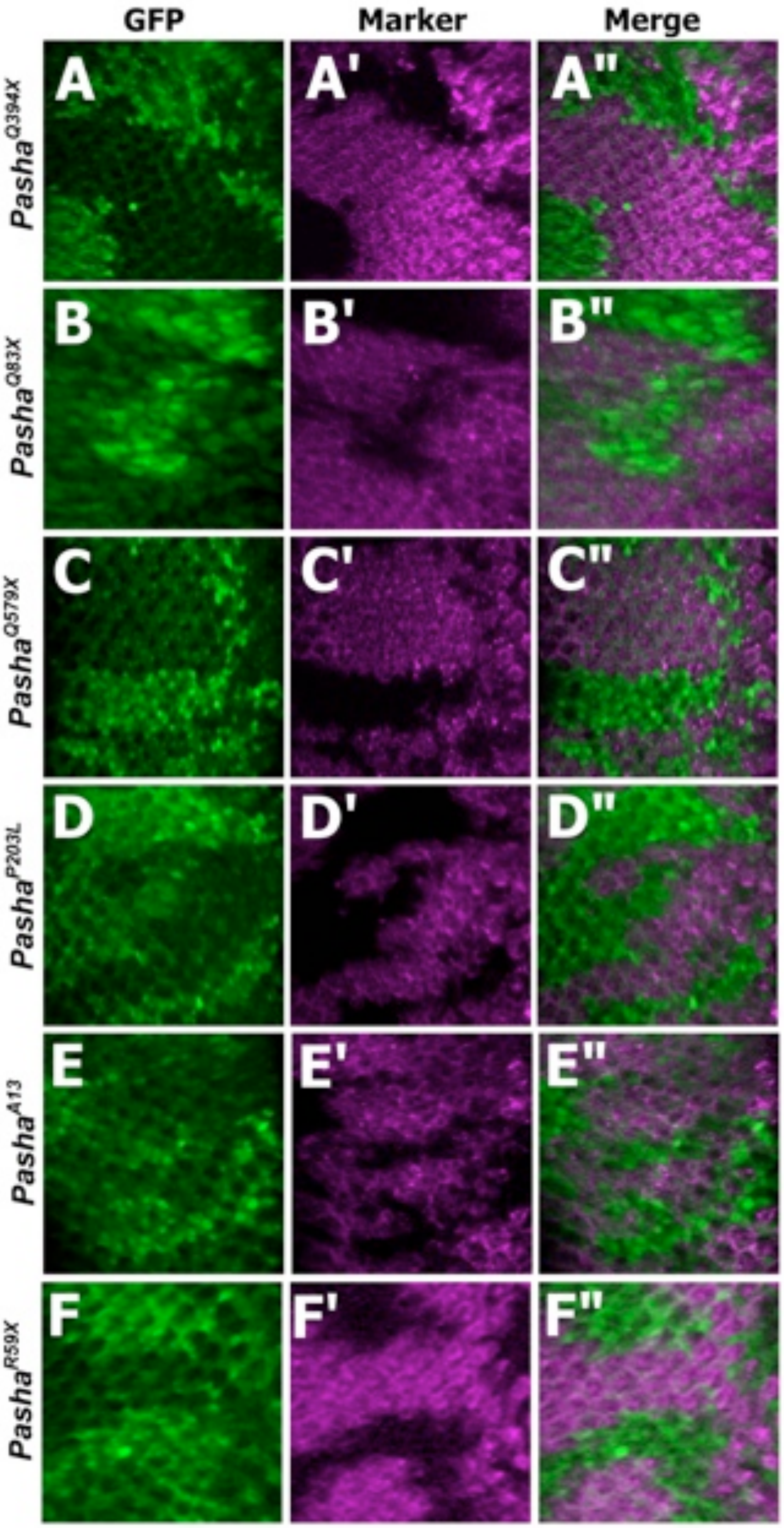

Figure S9. Expression of protein from *GMR>eGFP::Brd* (green) in mosaic larval eye discs containing clones of mutant cells homozygous for *Pasha* alleles *Q394X* (A), *Q83X* (B), *Q579X* (C), *P203L* (D), *A13* (E), and *R59X* (F). Mutant cells are marked by the absence of RFP protein (purple); cells with one or two copies of the wildtype *Pasha* allele express RFP.

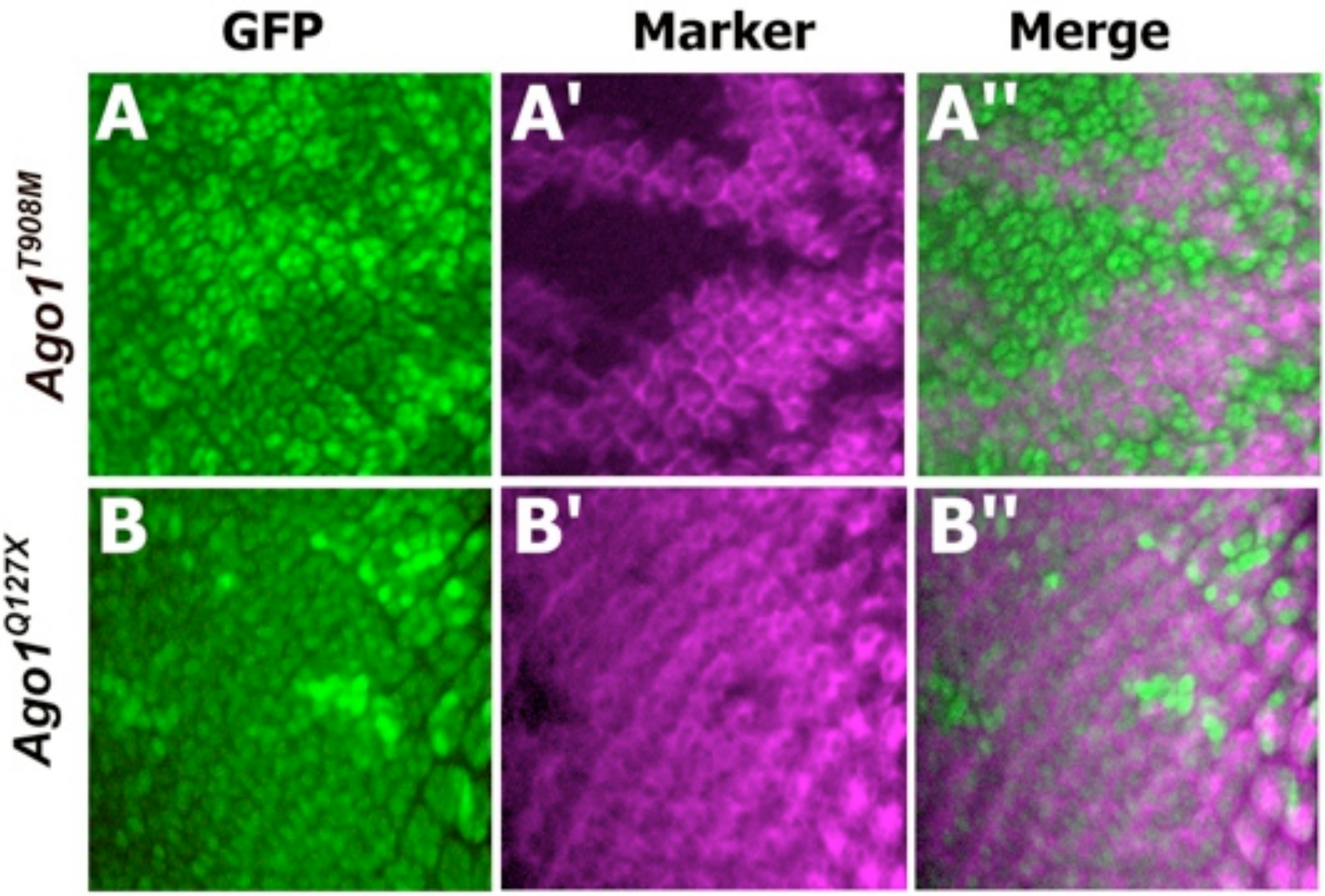

Figure S10. Expression of protein from *tub>eGFP::2x(miR-7)* (green) where two perfect binding sites for miR-7 are positioned in the 3'UTR. Mosaic larval eye discs contain clones of mutant cells homozygous for *Ago1* missense allele *T908M* (A) and nonsense allele *Q127X* (B). Mutant cells are marked by the absence of RFP protein (purple); cells with one or two copies of the wildtype *Ago1* allele express RFP.

Table S1. Lethal Phase Analysis of Zygotic Mutants. Allelic combinations are indicated. Numbers of animals scored at each stage of the life cycle are indicated.

Gene	Allele Combination	Embryos	Larvae	Pupae	Adults	% Hatch	% Pupate	% Eclose
**Wildtype**	**Ago1[T908M]/+**	308	258	148	132	83.766	48.052	42.857
**Ago1**	**R839X/Q127X**	700	0	0	0	0.000	0.000	0.000
**Ago1**	**R937C/Q127X**	291	32	0	0	10.997	0.000	0.000
**Ago1**	**E808K/Q127X**	500	185	1	1	37.000	0.200	0.200
**Ago1**	**D743N/Q127X**	500	274	73	55	54.800	14.600	11.000
**Ago1**	**J04/Q127X**	550	0	0	0	0.000	0.000	0.000
**Ago1**	**W894X/Q127X**	700	0	0	0	0.000	0.000	0.000
**Ago1**	**T908M/Q127X**	500	301	2	1	60.200	0.400	0.200
**Drosha Ago1**	**[Q884X] / + [Q127X] / +**	388	266	123	42	68.557	31.701	10.825
**Drosha**	**W1123X/Q884X**	234	121	61	0	51.709	26.068	0.000
**Drosha**	**Q938X/Q884X**	234	131	40	0	55.983	17.094	0.000
**Pasha**	**Q83X/Df**	245	148	104	0	60.408	42.449	0.000
**Pasha**	**R59X/Df**	224	111	74	1	49.554	33.036	0.446
**Pasha**	**Q579X/Df**	270	153	96	0	56.667	35.556	0.000
**Dicer-1**	**Q396X/Df**	211	82	47	0	38.863	22.275	0.000
**Dicer-1**	**G2035S/Df**	349	91	47	0	26.074	13.467	0.000
**Dicer-1**	**K43X/Df**	154	72	55	0	46.753	35.714	0.000
**Dicer-1**	**Q1233X/Df**	218	101	68	0	46.330	31.193	0.000
