# Supplementary material for: A Systematic Genetic Screen to Dissect the MicroRNA Pathway in Drosophila
Source: G3 (Bethesda). 2012 Apr 1;2(4):437–48. doi: 10.1534/g3.112.002030 (PMC3337472; doi:10.1534/g3.112.002030)
Supplement: Supporting Information [file supp_2.4.437_FigureS2.pdf]

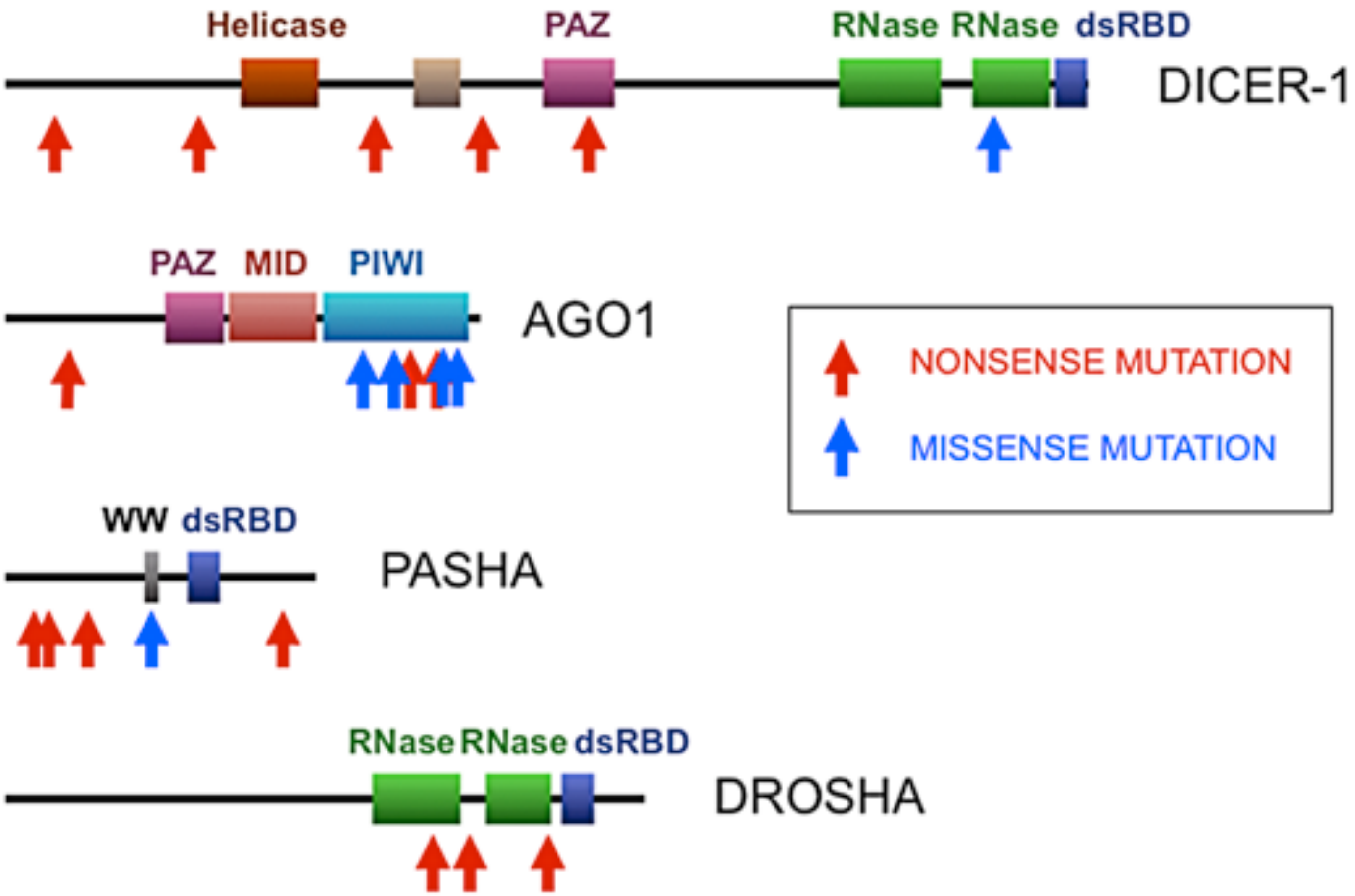


**Figure S2.** Schematic of Ago1, Dicer-1, Drosha, and Pasha polypeptides showing the conserved domains present in each protein. Indicated are the positions and of the various point mutations for each mutant allele that were isolated in the screen.
