# Supplementary material for: A Systematic Genetic Screen to Dissect the MicroRNA Pathway in Drosophila
Source: G3 (Bethesda). 2012 Apr 1;2(4):437–48. doi: 10.1534/g3.112.002030 (PMC3337472; doi:10.1534/g3.112.002030)
Supplement: Supporting Information [file supp_2.4.437_FigureS3.pdf]

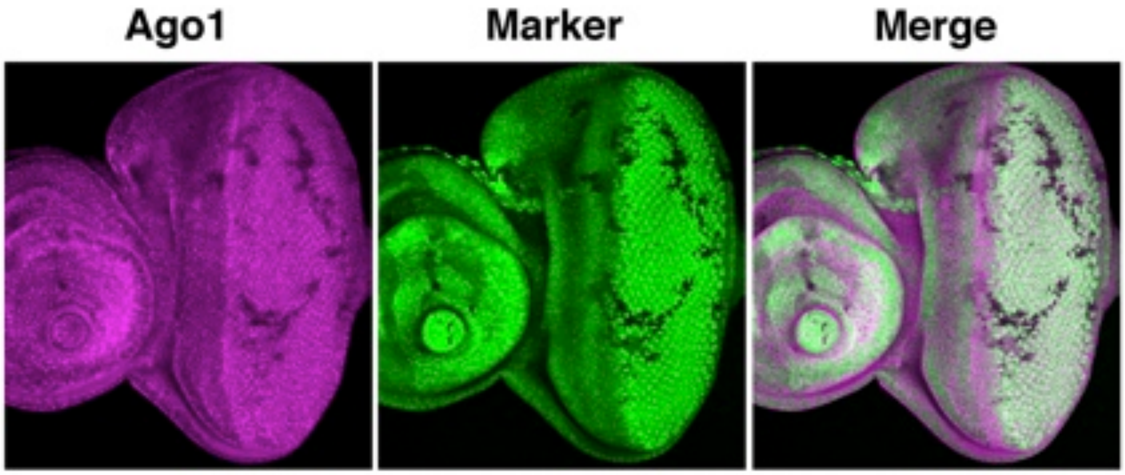

Figure S3. Ago1 protein (purple) stained with a monoclonal antibody in a larval eye disc containing clones of *Ago1*^{Q127X} mutant cells. These mutant cells are marked by the absence of a GFP marker (green); all GFP-positive cells contain one or two copies of the wildtype *Ago1* allele.
