# Supplementary material for: A Systematic Genetic Screen to Dissect the MicroRNA Pathway in Drosophila
Source: G3 (Bethesda). 2012 Apr 1;2(4):437–48. doi: 10.1534/g3.112.002030 (PMC3337472; doi:10.1534/g3.112.002030)
Supplement: Supporting Information [file supp_2.4.437_FigureS4.pdf]

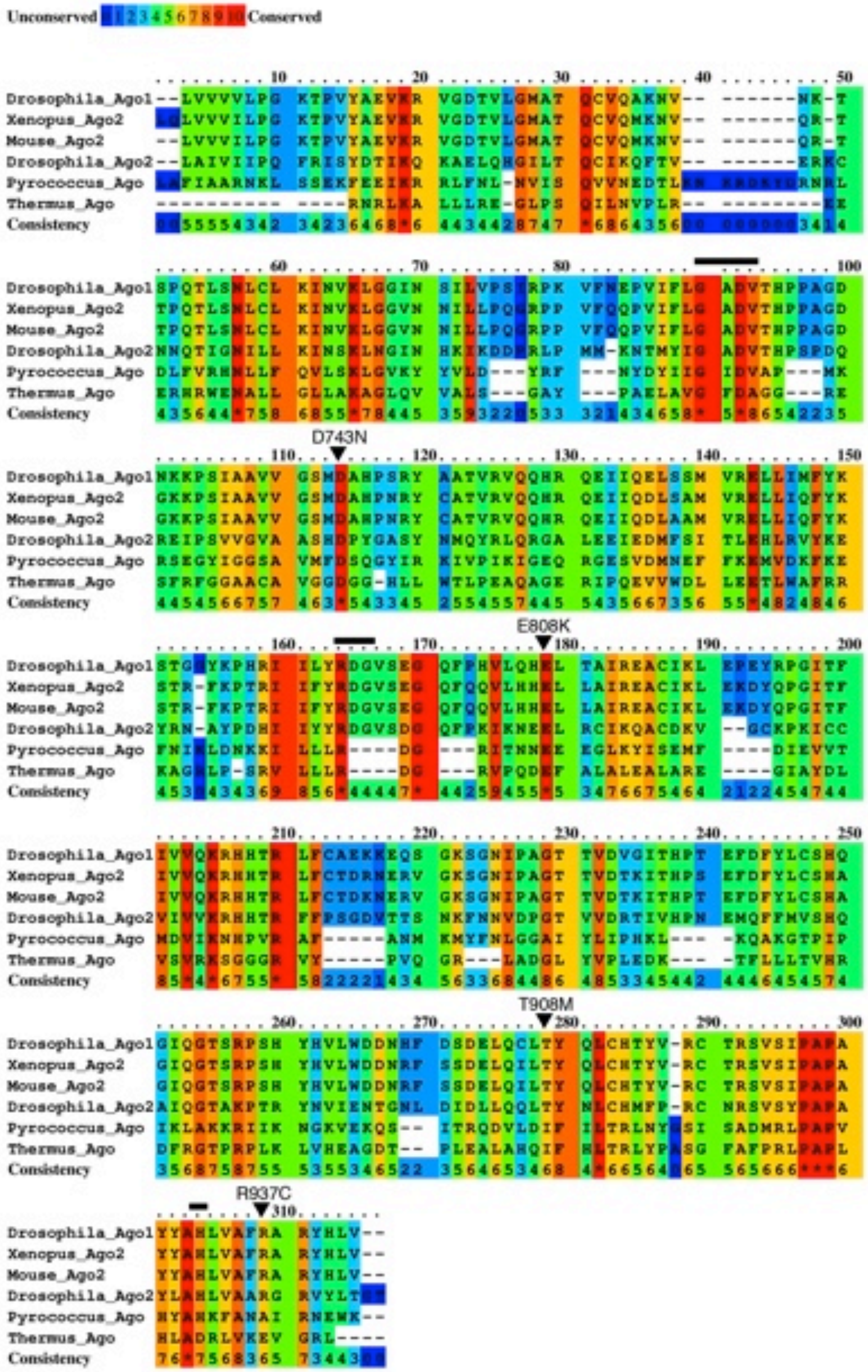

Figure S4. Alignment of Piwi domains from *Drosophila melanogaster* Ago1 and Ago2, *Xenopus laevis* Ago2, *Mus musculus* Ago2, archaebacterial *Pyrococcus fuiosus* Ago, and eubacterial *Thermus thermophilis* Ago. Color coding denotes degree of sequence conservation as indicated at top. Positions of the three clusters that coordinate the metal ion for RNA catalysis are indicated by black bars. Positions of the four residues mutated in missense Ago1 alleles are indicated by black triangles. Alignment was performed using the PRALINE algorithm.
