# Supplementary material for: A Systematic Genetic Screen to Dissect the MicroRNA Pathway in Drosophila
Source: G3 (Bethesda). 2012 Apr 1;2(4):437–48. doi: 10.1534/g3.112.002030 (PMC3337472; doi:10.1534/g3.112.002030)
Supplement: Supporting Information [file supp_2.4.437_FigureS5.pdf]

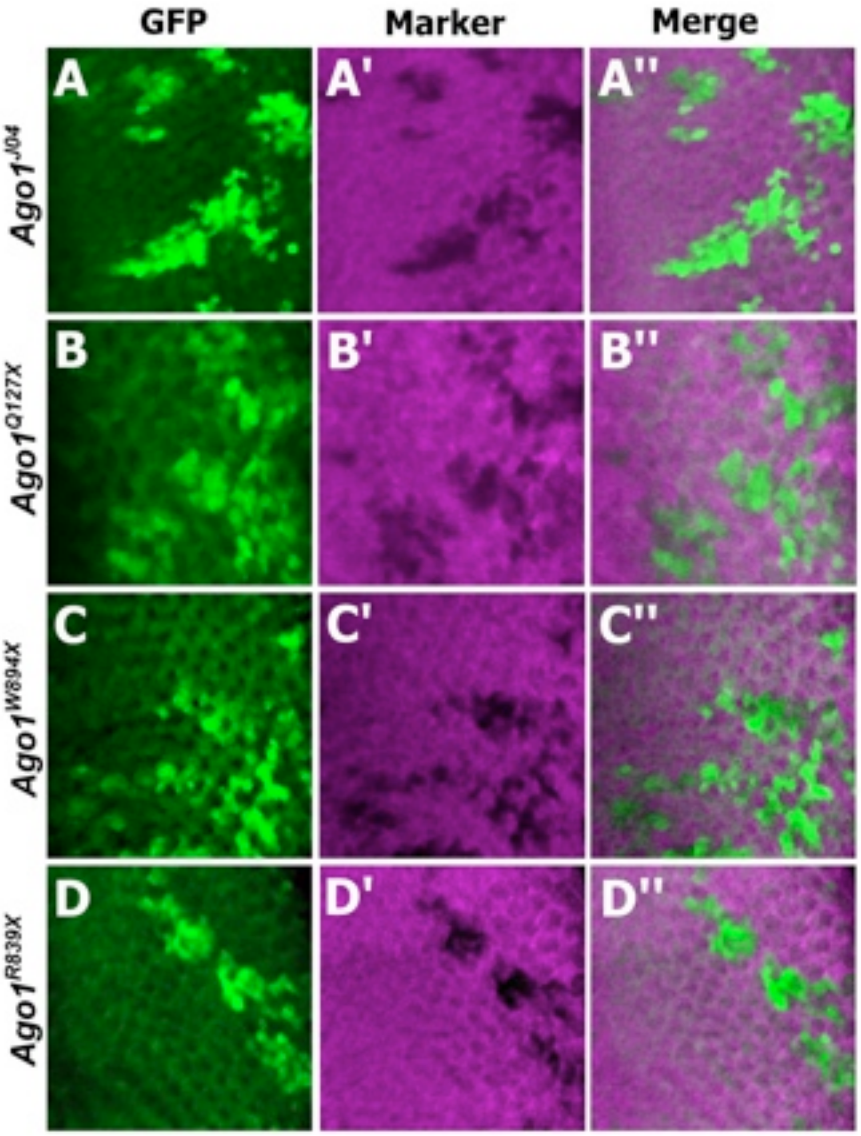

Figure S5. Expression of protein from *GMR>eGFP::Brd* (green) in mosaic larval eye discs containing clones of mutant cells homozygous for *Ago1* alleles *J04* (A), *Q127X* (B), *W894X* (C), and *R839X* (D). Mutant cells are marked by the absence of LacZ protein (purple); cells with one or two copies of the wildtype *Ago1* allele express LacZ.
