# Supplementary material for: A Systematic Genetic Screen to Dissect the MicroRNA Pathway in Drosophila
Source: G3 (Bethesda). 2012 Apr 1;2(4):437–48. doi: 10.1534/g3.112.002030 (PMC3337472; doi:10.1534/g3.112.002030)
Supplement: Supporting Information [file supp_2.4.437_FigureS8.pdf]

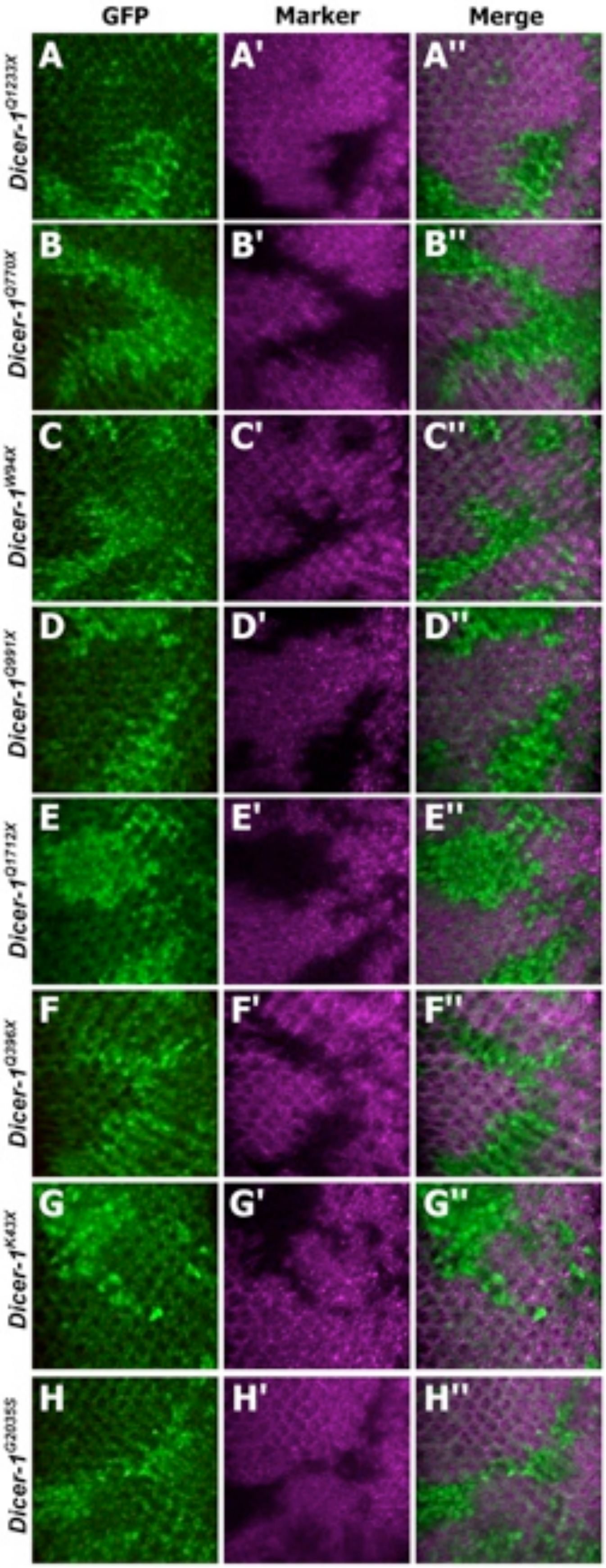

Figure S8. Expression of protein from *GMR>eGFP::Brd* (green) in mosaic larval eye discs containing clones of mutant cells homozygous for *Dicer-1* alleles *Q1233X* (A), *Q770X* (B), *W94X* (C), *Q991X* (D), *Q1712X* (E), *Q396X* (F), *K43X* (G), and *G2035S* (H). Mutant cells are marked by the absence of RFP protein (purple); cells with one or two copies of the wildtype *Dicer-1* allele express RFP.
