# Supplementary material for: A Systematic Genetic Screen to Dissect the MicroRNA Pathway in Drosophila
Source: G3 (Bethesda). 2012 Apr 1;2(4):437–48. doi: 10.1534/g3.112.002030 (PMC3337472; doi:10.1534/g3.112.002030)
Supplement: Supporting Information [file supp_2.4.437_FigureS9.pdf]

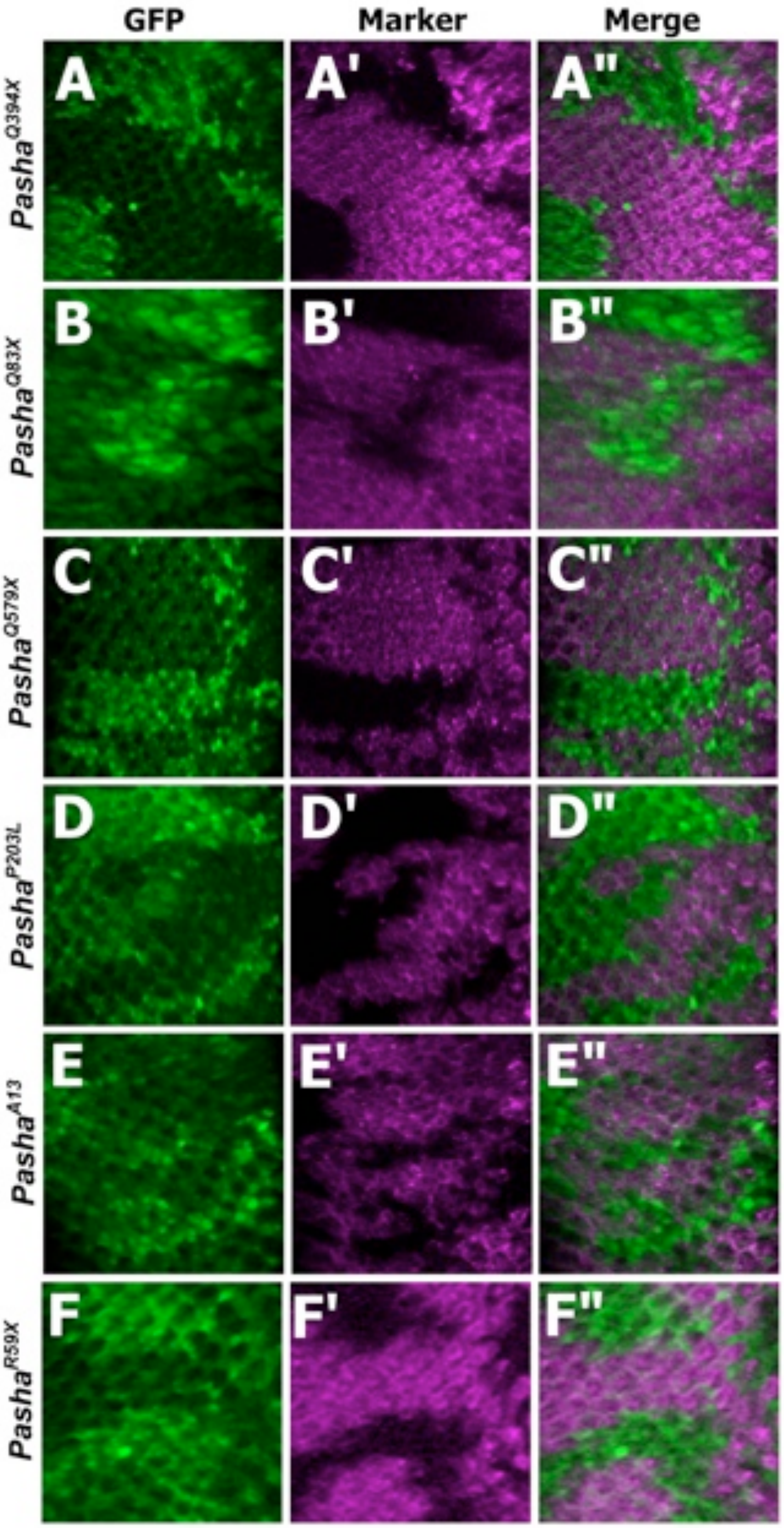

Figure S9. Expression of protein from *GMR>eGFP::Brd* (green) in mosaic larval eye discs containing clones of mutant cells homozygous for *Pasha* alleles *Q394X* (A), *Q83X* (B), *Q579X* (C), *P203L* (D), *A13* (E), and *R59X* (F). Mutant cells are marked by the absence of RFP protein (purple); cells with one or two copies of the wildtype *Pasha* allele express RFP.
