# Supplementary material for: A Systematic Genetic Screen to Dissect the MicroRNA Pathway in Drosophila
Source: G3 (Bethesda). 2012 Apr 1;2(4):437–48. doi: 10.1534/g3.112.002030 (PMC3337472; doi:10.1534/g3.112.002030)
Supplement: Supporting Information [file supp_2.4.437_FigureS10.pdf]

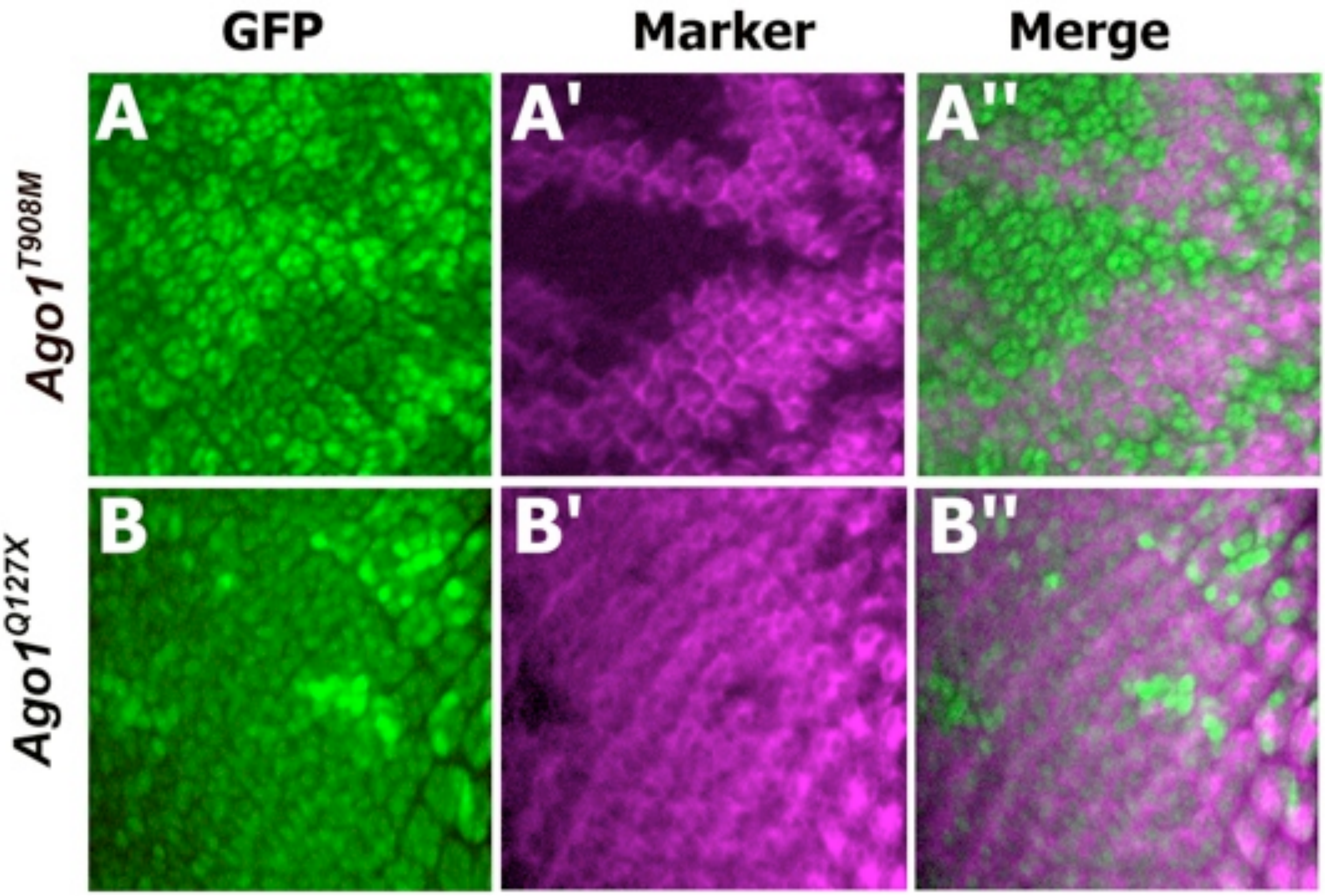

Figure S10. Expression of protein from *tub>eGFP::2x(miR-7)* (green) where two perfect binding sites for miR-7 are positioned in the 3'UTR. Mosaic larval eye discs contain clones of mutant cells homozygous for *Ago1* missense allele *T908M* (A) and nonsense allele *Q127X* (B). Mutant cells are marked by the absence of RFP protein (purple); cells with one or two copies of the wildtype *Ago1* allele express RFP.
