# Supplementary material for: A Systematic Genetic Screen to Dissect the MicroRNA Pathway in Drosophila
Source: G3 (Bethesda). 2012 Apr 1;2(4):437–48. doi: 10.1534/g3.112.002030 (PMC3337472; doi:10.1534/g3.112.002030)
Supplement: Supporting Information [file supp_2.4.437_TableS1.pdf]

Table S1. Lethal Phase Analysis of Zygotic Mutants. Allelic combinations are indicated. Numbers of animals scored at each stage of the life cycle are indicated.

Gene	Allele Combination	Embryos	Larvae	Pupae	Adults	% Hatch	% Pupate	% Eclose
**Wildtype**	**Ago1[T908M]/+**	308	258	148	132	83.766	48.052	42.857
**Ago1**	**R839X/Q127X**	700	0	0	0	0.000	0.000	0.000
**Ago1**	**R937C/Q127X**	291	32	0	0	10.997	0.000	0.000
**Ago1**	**E808K/Q127X**	500	185	1	1	37.000	0.200	0.200
**Ago1**	**D743N/Q127X**	500	274	73	55	54.800	14.600	11.000
**Ago1**	**J04/Q127X**	550	0	0	0	0.000	0.000	0.000
**Ago1**	**W894X/Q127X**	700	0	0	0	0.000	0.000	0.000
**Ago1**	**T908M/Q127X**	500	301	2	1	60.200	0.400	0.200
**Drosha Ago1**	**[Q884X] / + [Q127X] / +**	388	266	123	42	68.557	31.701	10.825
**Drosha**	**W1123X/Q884X**	234	121	61	0	51.709	26.068	0.000
**Drosha**	**Q938X/Q884X**	234	131	40	0	55.983	17.094	0.000
**Pasha**	**Q83X/Df**	245	148	104	0	60.408	42.449	0.000
**Pasha**	**R59X/Df**	224	111	74	1	49.554	33.036	0.446
**Pasha**	**Q579X/Df**	270	153	96	0	56.667	35.556	0.000
**Dicer-1**	**Q396X/Df**	211	82	47	0	38.863	22.275	0.000
**Dicer-1**	**G2035S/Df**	349	91	47	0	26.074	13.467	0.000
**Dicer-1**	**K43X/Df**	154	72	55	0	46.753	35.714	0.000
**Dicer-1**	**Q1233X/Df**	218	101	68	0	46.330	31.193	0.000
